# Tunable Lipid Coatings Enable Cytoplasmic siRNA Delivery by DNA Origami

**DOI:** 10.1021/acsami.6c03188

**Published:** 2026-06-17

**Authors:** Pauline B. M. Hendrickx, Anne des Rieux, Maartje M. C. Bastings

**Affiliations:** † Advanced Drug Delivery and Biomaterials, Louvain Drug Research Institute, 508610 UCLouvain, Brussels 1200, Belgium; ‡ Programmable Biomaterials Laboratory, Institute of Materials, Interfaculty Bioengineering Institute, School of Engineering, Ecole Polytechnique Fédérale Lausanne, Lausanne 1015, Switzerland

**Keywords:** interfering RNA, cytoplasmic delivery, DNA origami, lipid composition, endosomal escape

## Abstract

DNA origami nanostructures (DONs) offer precise architectural control for small RNA delivery, but their performance is limited by their susceptibility to nuclease degradation, weak cellular uptake, and inefficient endosomal escape. Here, we introduce an electrostatic lipid-coating strategy that introduces membrane-like properties to DONs while maintaining their structural integrity. By coincubating folded DONs with liposomes composed of a defined mixture of zwitterionic and cationic lipids, we identify a formulation window that yields colloidally stable, monodisperse lipid-coated DONs (LCDs). Systematic variation of the zwitterionic-to-cationic lipid ratio revealed that the fraction of cationic lipids strongly regulates the rate and extent of cellular uptake, with the 50 mol % cationic lipid content emerging as the most effective formulation. LCDs exhibited faster and more extensive cellular internalization and reduced early endosomal retention compared with PEG-coated DONs. The 50 mol % cationic formulation enabled efficient siRNA silencing comparable to benchmark lipid nanoparticles, underscoring the importance of rapid uptake and early endosomal escape for functional delivery while maintaining high cell viability. These findings establish an approach for integrating lipid functionalities onto DONs to improve cytoplasmic delivery while preserving full design flexibility.

## Graphical abstract

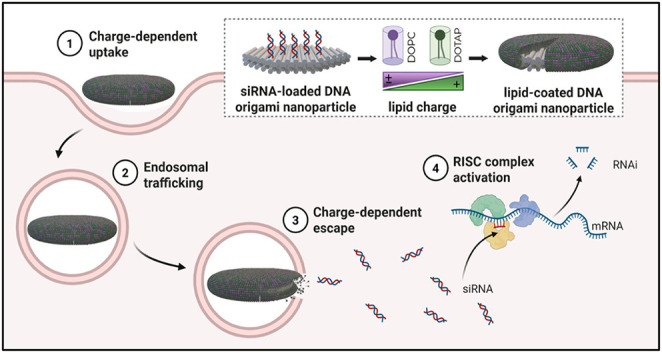

## Introduction

Delivering functional biomolecules directly to the cytoplasm remains one of the central challenges in nucleic acid therapeutics. Cytoplasmic access is essential for siRNA-mediated gene silencing, yet most nanocarriers are internalized through endocytosis and remain trapped in membrane-bound compartments.^1^ Nanoparticles offer unique opportunities to overcome these barriers by protecting fragile cargo, promoting cellular uptake, and enabling controlled endosomal escape through surface modulation.^2^ Among these, DNA origami nanostructures (DONs) provide atomically defined architectures that can be engineered with exceptional control over shape, mechanical properties, and surface presentation by folding a long ssDNA scaffold through strategic hybridization with multiple short ssDNA staple strands.^3,4^ Their programmable design supports precise spatial and stochastic addressability, allowing them to be used as a valuable tool for interfacing with or delivering therapeutics to biological systems.^5–9^ This structural precision has generated increasing interest in DONs as customizable platforms for nucleic acid delivery. Recent work further highlights their potential. Zhang et al. (2025)^32^ reported an inflammation-responsive DNA origami nanodevice enabling targeted siRNA delivery for the treatment of ulcerative colitis, demonstrating disease-specific activation and effective gene silencing. Such studies underscore the versatility of programmable DNA nanostructures for RNAi therapeutics. However, native DONs face significant biological limitations: their strong negative charge restricts membrane interaction and results in poor cellular uptake, endosomal escape is highly inefficient, and unprotected structures are rapidly degraded before reaching the cytoplasm.^10–12^ These constraints have hindered the translation of DONs toward therapeutic use and highlight the need for strategies that improve their stability and intracellular accessibility.

A variety of approaches have been explored to overcome the biological limitations of DONs, but each approach remains constrained by specific drawbacks. Oligolysine-PEG coatings have been widely used to enhance nuclease resistance, significantly improving their persistence under physiological conditions compared to noncoated structures.^13,14^ However, while they improve structural stability, there is limited evidence that they enhance specific cellular uptake or facilitate endosomal escape, and in some nanoparticle systems, PEGylation is known to reduce internalization, suggesting that careful surface design may be required to balance stability with efficient delivery.^15,16^ Endosomolytic peptides can facilitate cytosolic release but often induce aggregation and exhibit formulation-dependent toxicity.^17^ Cationic polymers increase uptake through electrostatic interactions, but their coatings are structurally heterogeneous, difficult to control, and associated with unpredictable cytotoxicity.^18,19^ Silica encapsulation provides strong structural protection while preserving particle addressability, but it has not been shown to enhance endosomal escape, limiting its suitability for cytoplasmic delivery.^20,21^ Attempts at lipid functionalization have been limited, typically relying on bulk lipid formulations lacking systematic evaluation of key parameters such as lipid composition, charge balance related to colloidal stability, or their protective properties toward nuclease activity.^22–25^ As a result, there is no robust existing strategy that achieves the combination of high uptake and efficient endosomal escape.

These limitations are further compounded by fundamental barriers associated with nucleic acid delivery. Therapeutic siRNA and miRNA require cytoplasmic access to exert their activity, yet naked RNA is rapidly degraded by ubiquitous ribonucleases and exhibits poor serum stability, leading to minimal cellular uptake.^26,27^ Following internalization, most delivery systems remain trapped in endosomal compartments, where acidification and enzymatic degradation prevent therapeutic efficiency.^1^ While conventional lipid-based nanocarriers improve internalization and endosomal escape, their performance is often still constrained by low cytoplasmic delivery of the therapeutic strand, dose-limiting toxicity, and inflammatory responses.^28–30^ Collectively, these challenges underscore the need for delivery systems that can protect nucleic acids, promote efficient uptake, and mediate reliable endosomal escape while maintaining their biocompatibility.

Lipid nanoparticles (LNPs) represent the current gold standard for therapeutic RNA delivery and have demonstrated high clinical efficacy, exemplified by the approved siRNA formulation Onpattro.^31^ Their performance comes from efficient nucleic acid encapsulation, strong cellular uptake, and moderately improved endosomal escape facilitated by ionizable lipids.^32^ However, LNPs are structurally heterogeneous and lack precise control over nanoscale architecture. Encapsulation occurs stochastically, resulting in variable loading stoichiometry and limited control over cargo distribution.^33^ Additionally, LNP formulations often show batch-to-batch variability and provide restricted opportunities for modular functionalization or controlled integration of multiple components.^34^ While these systems demonstrate high biological performance, this is typically accompanied by reduced structural precision. This consideration motivates the exploration of hybrid platforms that combine the functional benefits of lipid carriers with the structural programmability of engineered scaffolds, which may enable controlled stoichiometry, defined spatial organization of cargos (e.g., siRNA), and the modular incorporation of additional functionalities, such as targeting ligands.

In this work, we established a systematic screening and reproducible framework for generating lipid-coated DONs through electrostatic vesicle collapse. By tuning lipid composition and defining a charge-dependent formulation window, we identified conditions that prevent aggregation and maintain monodispersity. The resulting lipid-coated DONs were benchmarked directly against noncoated DONs, oligolysine-PEG-coated DONs, and clinically relevant LNPs, demonstrating significantly higher uptake and earlier endosomal escape compared to other DON coatings, with functional gene silencing efficiencies approaching those of LNPs. Together, the lipid-coated DONs present a platform that combines the biological performance of lipid nanoparticles with the structural precision, stoichiometric control, and modularity of DNA origami, overcoming the poor uptake and limited intracellular accessibility of native DONs while retaining their programmable architecture.

## Results and Discussion

### Defining the Formulation Window for Monodisperse Lipid-Coated DONs

A lipid coating offers two key advantages for DON-based delivery systems: it shields the DNA scaffold from nuclease-mediated degradation in physiological environments and provides membrane-like surface properties that enhance interactions with cellular membranes, thereby promoting both cellular internalization and endosomal escape. To exploit these benefits for improved cytoplasmic delivery, we developed a lipid-based coating strategy for DNA origami nanostructures. A compact, disk-shaped DON measuring 60 nm in diameter and 7 nm in height (Figure S1) was selected as the model architecture due to its proven structural stability and suitability for biological interfacing.^14,35^ This geometry served as the basis for engineering a lipid-coated DON platform optimized for efficient cytoplasmic access. To establish a robust coating method, we adopted an electrostatic coating approach using a defined 1:1 molar mixture of 1,2-dioleoyl-snglycero-3-phosphocholine (DOPC) and 1,2-dioleoyl-3-trimethylammonium-propane (DOTAP) lipids. DOPC was selected for its biocompatibility, zwitterionic character, and tendency to form stable bilayers that closely mimic those of natural membrane environments. DOTAP was included because of its structural similarity to DOPC and its cationic charge, enabling controlled modulation of surface electrostatics to enhance cellular internalization and promote fusion-driven endosomal escape.

Lipid vesicles were first extruded through a porous membrane, resulting in uniform liposomes measuring a diameter of 77.02 nm (±13.95) and a zeta potential of +43.9 mV (±2.9) (Figure S2). The liposomes were then incubated with prefolded DONs at room temperature under mechanical stress. Electrostatic interactions between the lipids and the negatively charged DNA, in the presence of monovalent and divalent cations, induced a spontaneous vesicle collapse around the DON surface, resulting in a lipid-coated DON (Figure 1A). To mitigate the pronounced aggregation tendency intrinsic to this highly charged system, a systematic screening of lipid-to-DNA ratios via a high-throughput DLS-based pipeline was performed (Figure 1B, S3). Aggregation was most prevalent at elevated DON concentrations or excessive lipid input. This behavior was consistent with charge inversion effects, where excess cationic lipid induces interparticle bridging and destabilization.^36,37^ The optimal formulation window was identified by mapping size distributions across DNA and lipid titrations, revealing a zone of minimal aggregation at lipid concentrations below 0.05 mg/mL.

**Figure 1:**
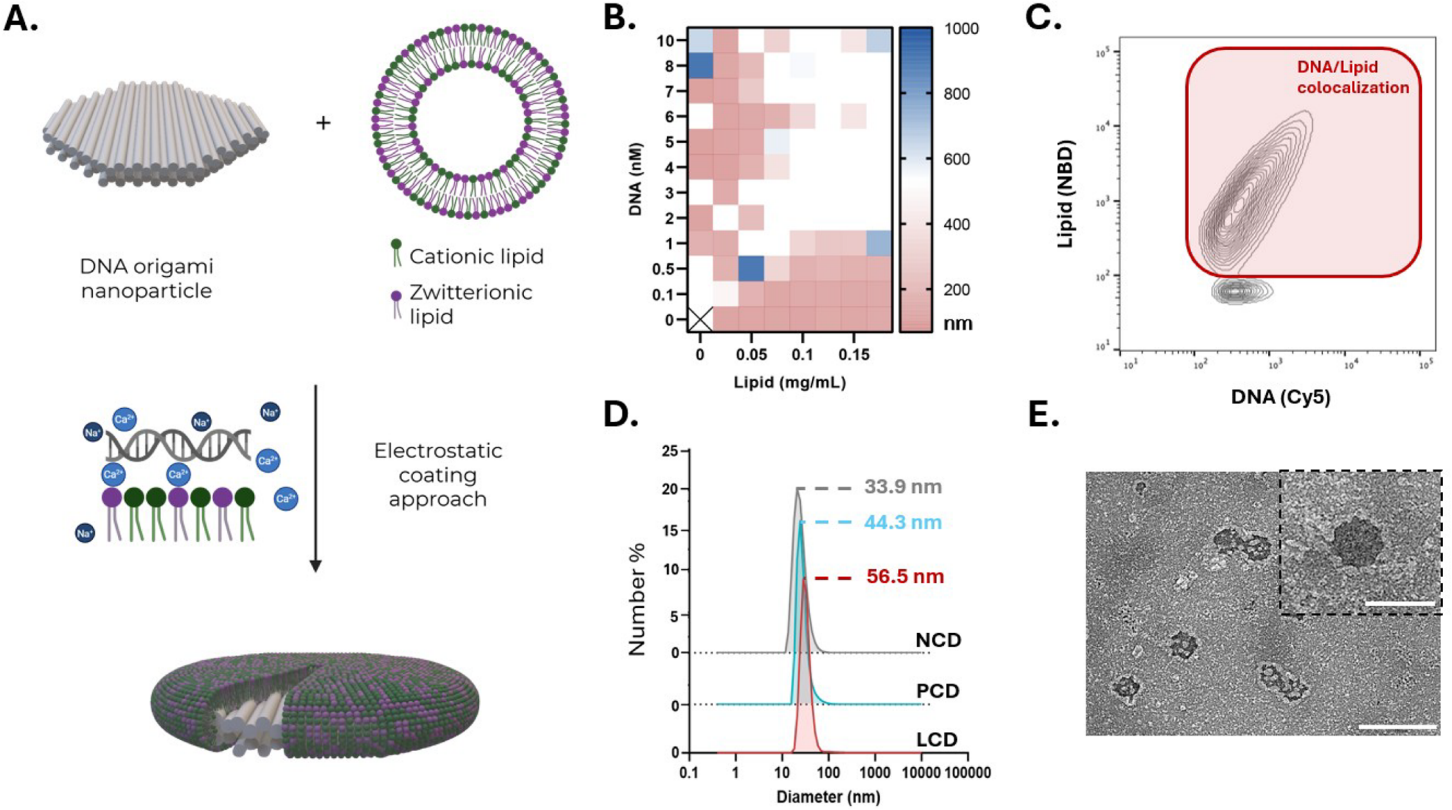
Development and optimization of a lipid-coating strategy for DNA origami nanoparticles (DONs). (A) Schematic illustration of the electrostatic lipid-coating approach. Preassembled DONs are coincubated with liposomes, where electrostatic interactions between the DNA backbone and lipid headgroups drive the formation of a lipid layer around the DON. Created in BioRender. Desrieux, A. (2026) https://BioRender.com/7wmyixa (B) High-throughput DLS-based screening of aggregation behavior during the lipid-coating process at varying lipid-to-DNA ratios (*N* = 2). (C) Quantification of lipid-DNA colocalization using nanoflow cytometry through Cy5-labeled DONs (5 nM) and NBD-labeled lipids (0.025 mg/mL) (*N* = 3). (D) Hydrodynamic size distribution of noncoated DON (NCD), PEG-coated DON (PCD), and lipid-coated DON (LCD). (E) Transmission electron microscopy (TEM) image of LCD. Scale bar: 100 nm (zoomed out) and 50 nm (zoomed in).

Quantitative colocalization analysis by nanoflow cytometry (nanoFCM) using Cy5-labeled DONs and NBD-labeled lipids indicated an increase in coating efficiency with increasing concentrations of DONs after multiple iterations (Figure S4). Finally, a high coating fidelity within this region, with 88.4% of particles exhibiting dual fluorescence signals, was achieved (Figure 1C). Consistent with this trend, agarose gel electrophoresis demonstrated the charge-neutralizing effect of the lipid layer: lipid-coated DONs showed markedly reduced electrophoretic mobility, which was restored upon treatment with chondroitin and Triton X-100, confirming the reversible electrostatic nature of the lipid–DNA interactions (Figure S5A). Zeta potential measurements further supported this behavior; coated particles exhibited a substantially lower positive ζ-potential than free liposomes, reflecting charge redistribution upon DNA–lipid association. In contrast, the highly negative charge of noncoated DONs caused rapid electrode discharge and cell failure during measurement, preventing successful acquisition (Figure S5B).

The selected formulation, 0.025 mg mL^–1^ lipid with 5 nM DONs, exhibited monodisperse morphology by TEM and minimal size heterogeneity by DLS (Figure 1D, E). However, when the DON concentration was kept constant and the lipid concentration was increased beyond this optimal ratio, the colloidal system destabilized, highlighting the necessity of precise charge balancing (Figure S6). This balance was achieved at an N:P ratio of ∼0.5, indicating that a stable coating can be achieved without requiring full charge neutralization of the DNA backbone. To rationalize why colloidal stability was achieved below full charge compensation, we considered the electrostatic profile of DONs under physiological ionic conditions. Approximating the structure as a uniformly charged disk and applying a linearized Poisson–Boltzmann framework, we obtained a Debye length of ∼0.84 nm, implying that only an outer shell of less than 1 nm contributes significantly to long-range interactions.^36,38,39^ In this geometry, this corresponded to roughly 20–30% of the phosphates being electrostatically “visible” to cationic lipids, which reduced the effective number of negative charges that must be compensated to avoid lipid-driven aggregation (Figure S7). This simple continuum estimate was consistent with the experimental work showing efficient coating of DNA origami at N:P ratios below formal charge neutralization.^40^

### Lipid Composition Tunes the Physicochemical Properties and Uptake of Lipid-Coated DONs

The lipid composition, and specifically the molar fraction of cationic lipids, is a key determinant of how a lipid bilayer interacts with both the DON surface and cellular membranes. Increasing the proportion of DOTAP enhances electrostatic interactions with the negatively charged DON, modulates membrane packing and fluidity, and adjusts the overall surface charge of the coated particle, factors known to influence cellular internalization and endosomal escape efficiency. To evaluate how these properties impact LCD performance, we systematically varied the DOPC:DOTAP molar ratio and examined the resulting physicochemical and biological behavior. Because DOPC and DOTAP share a similar cylindrical molecular geometry (*R*_0_ = 0), liposomes extruded from different compositions were expected to exhibit comparable curvature preferences (Figure 2A).^41,42^ Consistent with this expectation, DLS analysis showed minimal size variation across formulations (Figure 2B, C), indicating that overall vesicle morphology was preserved. In contrast, systematic titration of DOTAP from 0 to 50 mol % produced a progressive increase in zeta potential, from −9 mV to +45 mV, confirming controlled modulation of surface charge while preserving vesicle integrity (Figure 2B, C).

**Figure 2:**
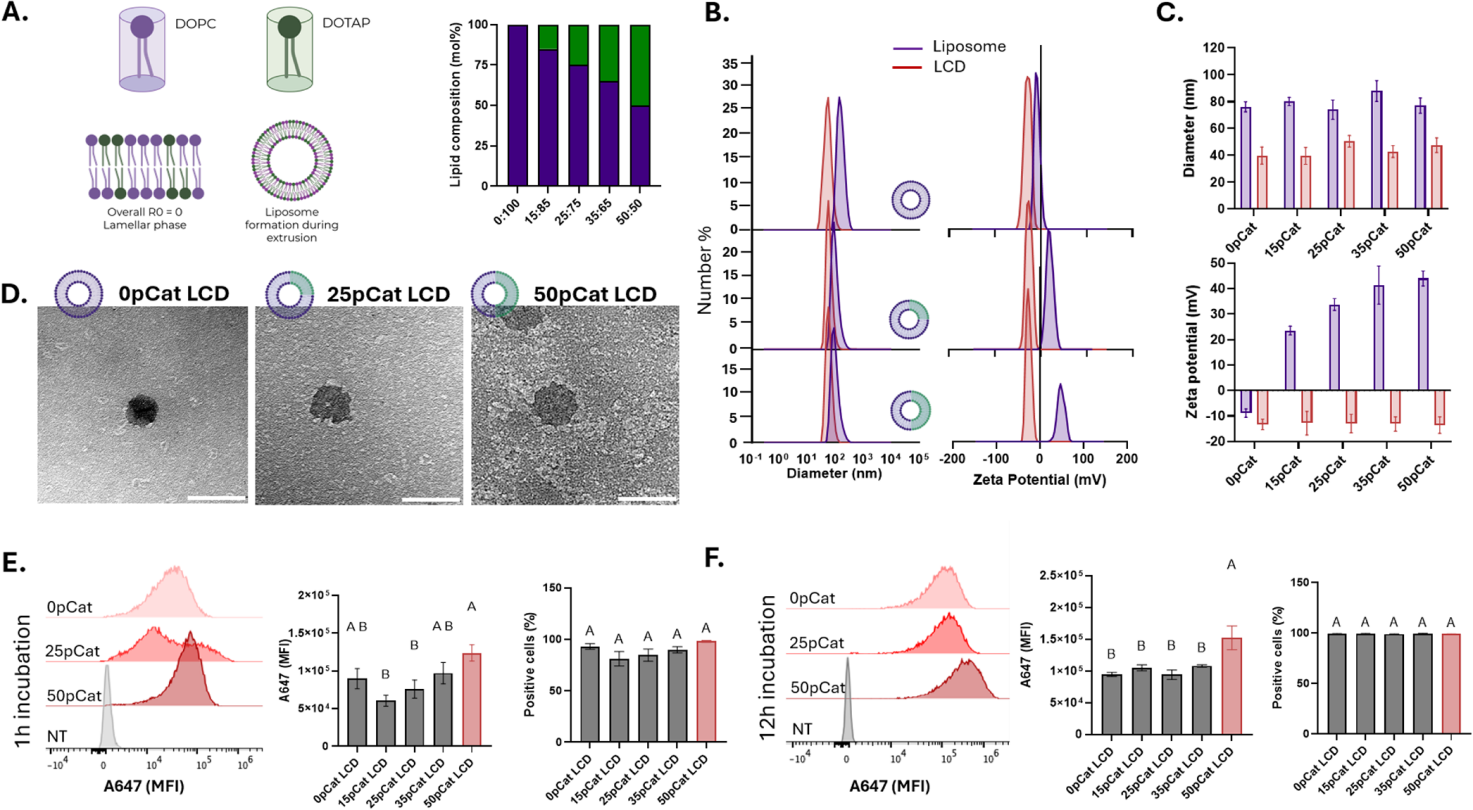
Influence of lipid composition on the physicochemical properties and cellular uptake of lipid-coated DONs. (A) Schematic representation of liposomes with varying DOTAP molar ratios (0%, 15%, 25%, 35%, and 50%) used for coating the DONs. Created in BioRender. Desrieux, A. (2026) https://BioRender.com/sm1jobp (B, C) Comparison of size and surface charge between liposomes and the corresponding LCDs. Measurements were performed at 5 nM DON concentration diluted in DMEM and 1x FoB buffer (*N* = 2). (D) TEM images of monodispersed LCDs with varying lipid compositions used for coating. Scale bar: 50 nm (E, F) Cellular uptake analysis of DONs labeled with Alexa Fluor 647 (A647) after 1 and 12 h incubation at 37 °C, showing the effect of lipid composition on internalization efficiency compared to nontreated (NT) cells (*N* = 3). Statistical analysis: One-way ANOVA followed by Tukey’s posthoc test. Conditions not sharing the same letters are significantly different.

Next, we applied each of the five liposome formulations to coat DONs by using the optimized coating protocol. All resulting LCDs remained colloidally stable, with comparable monodispersity and morphology confirmed by DLS and TEM (Figures 2C,D,S8). Despite the increasing DOTAP content in the liposomes, the ζ-potential of the LCDs remained largely unchanged across conditions (Figure 2C), and their charge neutralization observed with AGE was similarly unaffected (Figure S9). This likely reflects the preferential orientation of cationic DOTAP lipids toward the negatively charged DNA scaffold upon coating, effectively shielding the surface charge. However, lipid bilayers are inherently dynamic structures, and potential lipid flip-flop or lateral lipid redistribution may modulate surface presentation over time.^42,43^ Therefore, differences in cellular interaction profiles could still arise from compositional changes not reflected in the initial ζ-potential measurements.

Despite similar size and zeta potential across LCDs, uptake profiles varied notably with lipid composition. To compare these formulations in a sensitive and reproducible model, we used BV2 microglial cells, whose high endocytic activity makes them well-suited for evaluating small interfering RNA delivery. We incubated the various LCD formulations with BV2 cells for 1 h and quantified internalization by flow cytometry following nuclease treatment to remove all extracellular signals (Figure S10A). Among all tested conditions, the 50 mol % DOTAP (50pCat) LCD exhibited the highest uptake (Figure 2E, F). Although ζ-potential measurements did not reveal substantial differences between formulations, this does not preclude composition-dependent variations in the local presentation of cationic moieties and their resulting membrane interactions, in line with previous reports showing enhanced internalization of cationic nanoparticles through electrostatic interactions with anionic cell membranes.^44,45^ Interestingly, the 25 mol % DOTAP formulation (25pCat) showed a bimodal uptake distribution at 1 h, indicative of early heterogeneity that diminished with longer incubation, consistent with progressive uptake kinetics. By contrast, intermediate formulations (15pCat and 25pCat) displayed markedly lower cellular uptake than anticipated based on their DOTAP content, representing an unexpected deviation from a simple composition-uptake trend. This observation suggests that the relationship between lipid composition and uptake efficiency may be nonlinear. While the underlying mechanism was not directly investigated here, we hypothesize that formulation-dependent differences in lipid packing and headgroup geometry could influence membrane fluidity and interfacial organization, thereby modulating particle-cell interactions. In addition, the interplay between lipid fluidity and surface charge is known to affect protein corona formation on lipid-based nanoparticles, which may further contribute to the observed uptake variation.^46^ After 12 h of incubation, the differences in uptake became less pronounced, although the 50pCat LCD consistently outperformed all other formulations. The rapid internalization observed for the 50pCat LCD is particularly relevant for nucleic acid-based therapeutics. Given the inherent instability of nucleic acids in physiological environments, fast uptake significantly increases the likelihood of preserving therapeutic integrity and function.^47^

To determine whether DOTAP content also modulates intracellular trafficking, we assessed endosomal retention by confocal microscopy and Manders coefficient analysis after 6 h of incubation (Figure S11). Colocalization of LysoTracker Green staining with Alexa647-labeled DONs was quantified, and while all LCDs containing DOTAP exhibited reduced overlap with lysosomal signals relative to the DOPC-only condition (0pCat), no clear trend was observed across increasing DOTAP concentrations (Figure S10B). This indicates that the presence, but not necessarily the quantity, of cationic lipids promoted endosomal escape. These findings suggest that the inclusion of cationic lipids facilitated improved cytoplasmic access, but further increases in DOTAP content did not proportionally enhance endosomal release under the tested conditions.

### Comparative Analysis of Intracellular Trafficking and Uptake Kinetics across Coating Strategies

To benchmark the performance of LCDs against those of existing delivery vehicles, we compared their intracellular trafficking and uptake kinetics to those of PCDs, NCDs, and standard LNPs in BV2 microglial cells. The LNPs, with a lipid composition containing an ionizable lipid (D-Lin-MC3-DMA), cholesterol, DSPC, and DSPE-PEG2000 at a molar ratio of 50:38.5:10:1.5, were used due to their well-studied properties and clinical use for the delivery of RNA-based drugs.^31^ These formulations were selected to represent a spectrum of surface chemistries, allowing a direct comparison of the physicochemical influence on cellular processing. In addition to evaluating intracellular behavior, we assessed the stability of NCDs, PCDs, and LCDs to determine how each coating influences resistance to degradation prior to cellular uptake. As expected, NCDs are less resistant to nuclease-induced degradation and serum-induced degradation compared with PCDs and LCDs, indicating that coating-dependent stability may influence particle internalization and subsequent intracellular trafficking (Figure S12).

Endosomal retention was first assessed by confocal microscopy using LysoTracker Green to stain lysosomal compartments. Co-localization with A647-labeled nanostructures was quantified using the Manders coefficient at 1-h and 6-h postincubation. After 1 h, all nanoparticle types exhibited Manders coefficients above 0.5, indicating that most of the internalized material remained within endolysosomal compartments (Figure 3A). Notably, the LCDs showed a significantly lower Manders coefficient than PCDs, suggesting that the lipid coating facilitated more efficient early endosomal escape (Figure 3C). This early escape profile aligns with the known capacity of cationic lipids, such as DOTAP, to destabilize endosomal membranes, thereby accelerating cytoplasmic delivery, which is essential for nucleic acid therapeutics given their susceptibility to degradation in acidic, enzyme-rich compartments.^48^

**Figure 3:**
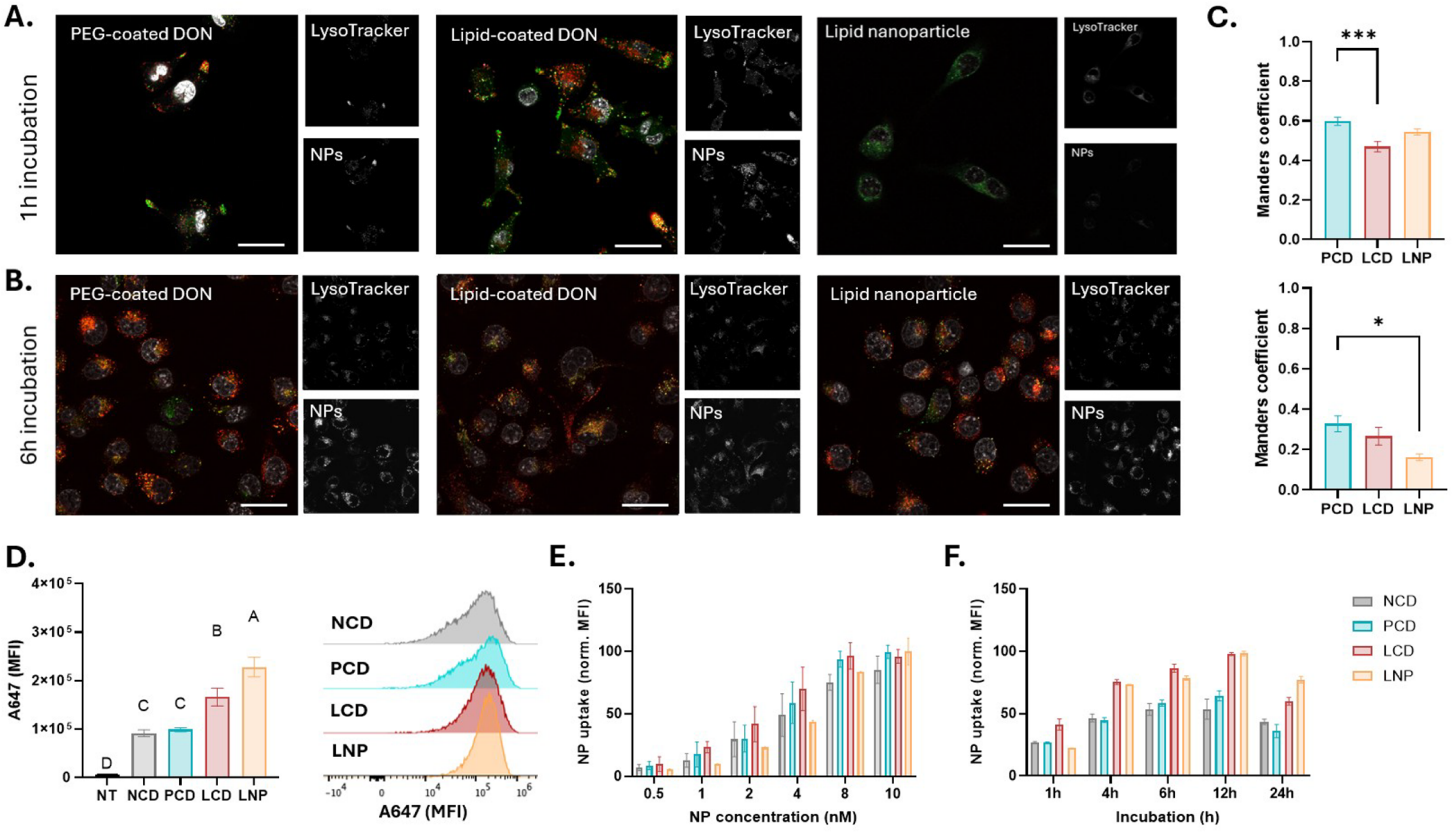
Cellular uptake and endosomal retention of differently coated DONs compared to lipid nanoparticles (LNPs). (A, B) Confocal fluorescence microscopy images showing the internalization of nanoparticles (NPs) in BV2 microglial cells after 1 h (top) and 6 h (bottom) of incubation. NPs were visualized through A647-labeled DNA (red), nuclei stained with Hoechst (gray), and acidic compartments stained with LysoTracker Green (green). Scale bar: 20 μm (C) Endosomal retention was quantified using CellProfiler by calculating Mander’s coefficient between A647 (DON) or Cy5 (LNP) and LysoTracker signals (*N* = 2). (D) Flow cytometry-based quantification of NP uptake after 12 h incubation by BV2 microglial cells (*N* = 3). Statistical analysis: One-way ANOVA followed by Tukey’s posthoc test. Conditions not sharing the same letters are significantly different. (E) Dose-response curves of NP uptake after 12 h incubation at varying NP concentrations. Data were normalized to the highest uptake values for DON-based (A647) and LNP (Cy5) samples (*N* = 2). (F) Kinetic analysis of NP uptake over time at 2 nM concentration, normalized to the maximum uptake values of DON-based (A647) and LNP (Cy5) samples (*N* = 2).

After 6 h of incubation, the Manders coefficient decreased for all formulations, consistent with time-dependent endosomal processing and escape (Figure 3B,C).^1^ Here, LNPs demonstrated the lowest degree of lysosomal colocalization, significantly outperforming PCDs. While LCDs continued to show reduced retention compared to PCDs, the difference was not statistically significant at this later time point, suggesting that LCDs promote more efficient early-stage escape compared to the PCDs. To assess cellular universality, endosomal escape was additionally evaluated in primary mixed glial cultures encompassing microglia, astrocytes, and oligodendrocyte precursors. The trends observed across all formulations were consistent with those obtained in BV2 cells, supporting the generalizability of the endosomal trafficking behavior across CNS cell types (Figure S13A,B).

To evaluate the overall cellular uptake, we next performed quantitative flow cytometry using fluorescently labeled DONs and LNPs. LCDs achieved significantly higher uptake than both NCDs and PCDs, consistent with prior observations of enhanced membrane interaction driven by cationic lipid content (Figure 3D).^49,50^ LCDs also approached uptake levels of benchmark LNPs, although the internalization efficiency of LNPs remained superior across the tested formulations. Across the tested formulations, NCDs and PCDs exhibited similar uptake profiles. These findings underscore the potential of LCDs to match the internalization efficiency of the established delivery vehicles. Validation in primary mixed glial cultures corroborated these results, with cellular uptake trends across all formulations remaining consistent with those observed in the BV2 cells. Notably, LCDs demonstrated slightly higher uptake than LNPs specifically in the microglia of this primary model, although this difference did not reach statistical significance (Figure S13C).

The analysis of uptake as a function of dose revealed distinct kinetic regimes. DON-based nanostructures showed saturable uptake behavior, which plateaued at higher concentrations, indicative of receptor-limited or energy-dependent internalization pathways. In contrast, LNPs showed a more linear dose–response profile, suggesting ongoing uptake capacity over the tested concentration range (Figure 3E). Time-course analysis showed that most formulations reached peak internalization within 12 h, followed by a modest decline at longer incubation times, likely reflecting intracellular processing or exocytosis, often seen in similar kinetic assays (Figure 3F).^51^

In summary, LCDs outperformed NCDs and PCDs in both uptake kinetics and endosomal escape efficiency, while LNPs retained an overall advantage in total cellular accumulation. These results highlight the value of lipid functionalization in tuning DNA origami interfaces for improved biological delivery and position LCDs as a promising strategy to bridge the structural programmability of DNA origami with improved cellular delivery characteristics.

### siRNA Loading, Release, and Gene Silencing Efficiency of Lipid-Coated DONs

To assess the therapeutic potential of lipid-coated DNA origami nanostructures (LCDs) for the cytoplasmic delivery of small interfering RNA (siRNA), we evaluated their siRNA loading efficiency, release kinetics, and gene silencing performance in vitro, using benchmark lipid nanoparticles (LNPs) and alternative DON coatings as comparators.

DONs were functionalized with siRNA through a modular strand extension strategy, in which each siRNA molecule was linked via a complementary antihandle strand (Figure S14A, B). The programmable nature of the attachment sites enables precise stoichiometry control over the siRNA loading, which was confirmed with a qualitative agarose gel and a quantitative qPCR assay (Figure S14C). This architecture enables siRNA hybridization through Watson–Crick base pairing and allows intracellular reductive cleavage of an incorporated disulfide bond in response to cytoplasmic glutathione (GSH) levels, triggering release at the site of action (Figure 4A). Agarose gel electrophoresis confirmed efficient siRNA incorporation and rapid release under reducing conditions, with nearly complete strand displacement observed after 1 h in 10 mM GSH, mimicking intracellular redox conditions (Figure 4B).^52^

**Figure 4:**
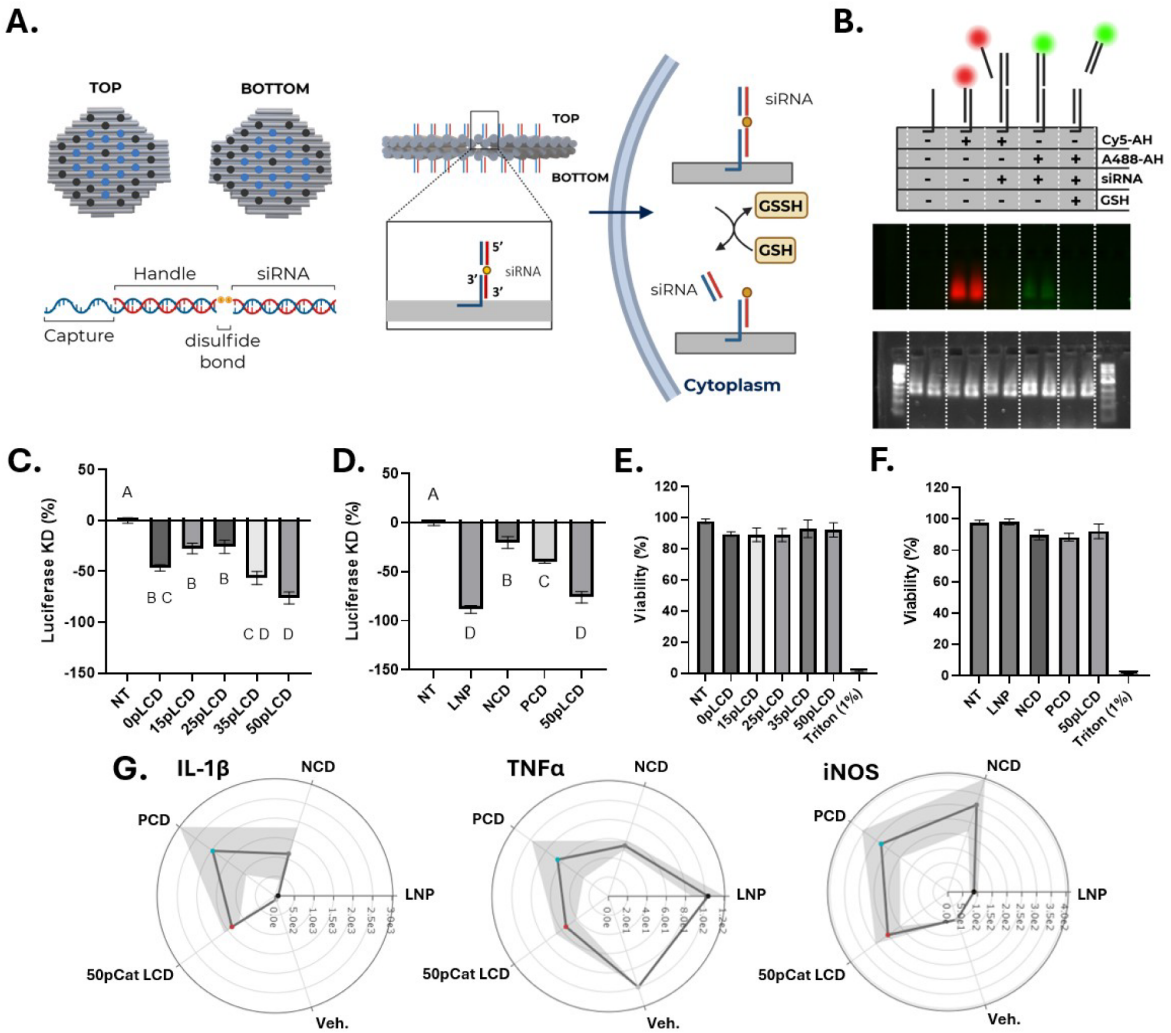
siRNA Loading, Release, and Gene Silencing Efficiency of Lipid-Coated DONs. (A) Schematic of siRNA loading and release mechanism onto the DON. Created in BioRender. Desrieux, A. (2026) https://BioRender.com/0wp18ca. (B) Characterization of siRNA loading and reductive release from DONs by 1% agarose gel electrophoresis. siRNA incorporation was quantified using a Cy5-labeled antihandle strand hybridized to DON-extended handles. Reductive release was evaluated under 10 mM glutathione (GSH) using an Alexa Fluor 488 (A488)-labeled guide strand complementary to the antisense siRNA strand (*n* = 2). (C) Luciferase knockdown efficiency after 24 h incubation at 37 °C with 40 nM siRNA. Effect of varying lipid-coating ratios on silencing efficiency (D) and a comparison between differently coated DONs and siRNA-loaded LNPs, normalized to vehicle control. (E, F) Cell viability after 24 h incubation under the same conditions as the knockdown assay, demonstrating cytocompatibility of DON-based formulations. (G) Inflammatory response induced after incubation with the different nanoparticle formulations, quantified by qPCR analysis and normalized to set the vehicle to 100 (Veh.) (*N* = 3). Statistical analysis: One-way ANOVA followed by Tukey’s post hoc test.

To evaluate gene silencing performance, DONs loaded with antiluciferase siRNA were incubated with luciferase-expressing BV2 microglial cells for 24 h. Among DON formulations, the 50pCat LCD achieved the most potent knockdown, significantly outperforming both the 15pCat and 25pCat LCDs (Figures 4C and S15). This trend mirrors the early uptake kinetics observed in flow cytometry (Figure 2F), suggesting that not only the magnitude of cellular uptake but also the speed of internalization and subsequent endosomal escape are critical determinants of siRNA efficacy. Given the rapid degradation of siRNA in intracellular compartments, these findings underscore the importance of kinetic delivery for maintaining nucleic acid bioactivity. In direct comparison to benchmark LNPs, the 50pCat LCD exhibited comparable silencing efficiency and significantly outperformed both NCDs and PCDs (Figures 4D and S15). These results highlight the capacity of lipid-functionalized DONs to mediate efficient siRNA delivery.

Cellular viability following particle exposure was assessed via a PrestoBlue metabolic activity assay. All DON-based formulations, including LCDs, maintained cell viability above 80%, with only minor differences across coating types (Figure 4E,F).^53^ These values are consistent with the established biocompatibility thresholds for nanoparticle delivery systems. A reduction in metabolic activity was observed in the Triton X-100-treated control, validating the assay’s sensitivity under cytotoxic conditions.

To further examine immunological responses to DON formulations, we quantified the expression of key pro-inflammatory markers, TNFα, IL-1β, and inducible nitric oxide synthase (iNOS), via qPCR following 24-h incubation. While TNFα and IL-1β levels remained within an acceptable range for all DON formulations, iNOS expression was markedly elevated, reaching a 10-fold increase relative to untreated controls (Figure 4G), though the associated NO production remained subcytotoxic, with cell viability assays confirming no significant cytotoxicity across the tested conditions. This pattern is consistent with activation of intracellular DNA-sensing pathways such as TLR9 or the cGAS–STING axis, both of which are known to induce iNOS upregulation in response to cytoplasmic DNA,^54^ with potential downstream consequences for intracellular trafficking that remain to be directly investigated. Interestingly, the 0pCat LCD induced minimal iNOS expression (Figure S16), suggesting that either the absence of cationic lipid reduces immunostimulatory potential or that these particles are released into the cytoplasm of the cell to a lesser extent, as indicated in (Figure S10B). The cationic lipids used in LCDs have previously been implicated in inflammatory responses, raising the possibility that charge-driven interactions with innate immune receptors contribute to the observed iNOS induction.

## Conclusion

Through an electrostatic coating approach, we introduce a lipid-based strategy that merges the structural precision of DNA origami with membrane-like functionalities essential for cytoplasmic delivery. This work defines a formulation window in which DONs can be reproducibly coated with lipids while maintaining colloidal stability, mitigating long-standing challenges of nuclease degradation and inefficient endosomal escape. By exploiting controlled electrostatic interactions rather than covalent modification, the coating preserves the intrinsic programmability of the DNA scaffold while providing biologically relevant surface properties, including enhanced membrane affinity and tunable surface charge, which together influence endosomal escape and intracellular trafficking.

Our results indicate that full-charge neutralization is not required for colloidal stability or efficient coating. Instead, compensating for only a fraction of surface-accessible phosphates is sufficient to achieve robust lipid wrapping. This insight explains why stable LCDs form at low N:P ratios and provides a quantitative design principle for future lipid-DNA assemblies. Moreover, systematic tuning of the DOPC:DOTAP ratio revealed that cationic lipids primarily modulate uptake kinetics rather than bulk physicochemical properties, underscoring the importance of early trafficking events for nucleic acid efficacy.

Functionally, LCDs displayed enhanced cellular internalization and reduced early endosomal retention relative to PEG-coated DONs, achieing delivery efficiencies approaching those of clinically validated LNPs. The strong siRNA silencing achieved with the 50 mol % DOTAP LCD highlights the significance of rapid uptake and early escape as determinants of functional performance. While LNPs still achieved the highest overall accumulation, LCDs reached comparable functional potency while offering precise geometric control and modular loading, which are not accessible with conventional lipid nanoparticles.

Together, these findings establish a foundational strategy for integrating lipid functionalities onto DONs to enable efficient cytoplasmic delivery while maintaining full design flexibility. Beyond therapeutic small RNA delivery, this platform opens new possibilities for mechanistic studies of particle-level RNA dosing, an area fundamentally obscured by the inherently heterogeneous LNP formulation, and provides a basis for programmable lipid-DNA nanoparticles.

## Experimental Section

### DNA Origami Folding and Purification

The M13mp18-based p7560 DNA scaffold (Tilibit) and sequence-specific staple strands (Integrated DNA Technologies), listed in Table S1 were used for all DNA origami assemblies as previously described.^35^ Briefly, prior to folding, the scaffold was subjected to four sequential endotoxin removal cycles consisting of Triton X-114 (ThermoFisher) phase separation followed by sedimentation. DNA origami nanostructures (DONs) were assembled by combining 10 nM scaffold with a 10-fold molar excess of nonmodified staple strands and a 5-fold molar excess of fluorophore-labeled (AF647 or Cy5) strands, if specified for the experiment, in FoB buffer (5 mM Tris, 1 mM EDTA, 5 mM NaCl, 18 mM MgCl_2_, pH 8). The total reaction volume was 75 μL. Mixtures were annealed in a Biometra TRIO thermocycler using the following program: 80 °C for 5 min, followed by a linear cooling ramp from 60 to 20 °C at −1 °C/h. Assembled DONs were purified via polyethylene glycol (PEG) precipitation. Annealing mixtures were combined in a 1:1 (v/v) ratio with 2× PEG precipitation buffer (15% PEG8000, 0.5 M NaCl, 5 mM Tris, 1 mM EDTA, 18 mM MgCl_2_) and incubated at room temperature for 30 min, rotating. Solutions were centrifuged at 16,000 × g for 40 min at 20 °C, after which the supernatant was removed, and the DON-containing pellets were immediately resuspended in FoB buffer. Final DON concentrations were quantified by UV absorbance at 260 nm using a NanoDrop spectrophotometer. Purified samples were stored at 4 °C for short-term use or at −80 °C for long-term preservation.

### Liposome Preparation and Extrusion

1,2-Dioleoyl-3-trimethylammonium-propane (DOTAP) and 1,2-dioleoyl-*sn*-glycero-3-phosphocholine (DOPC) lipids, both at a stock concentration of 25 mg/mL (Avanti Polar Lipids), were used for all liposome preparations. One mol % PE-NBD lipids were added to liposome preparations used for NanoFCM experiments. Lipid mixtures were prepared according to the molar compositions specified for each experiment, ranging from 0 to 50 mol % of DOTAP. Chloroform-dissolved lipids were transferred to a round-bottom flask, and the solvent was removed under a continuous argon stream, followed by incubation under vacuum conditions (800 Pa, 30 min) to form a dry lipid film. Lipid films were rehydrated in 0.1× FoB buffer to a final lipid concentration of 1 mg/mL. Suspensions were vortexed thoroughly to ensure complete detachment of the lipid film from the flask surface. The resulting multilamellar lipid suspensions were extruded using an Avanti Mini-Extruder equipped with 200 nm polycarbonate membranes (Avanti, item no. 10417004). Samples were passed through the membrane 20 times to obtain unilamellar vesicles. For characterization, liposomes were diluted 1:50 in 0.1× FoB for hydrodynamic size and polydispersity index (PDI) measurements via dynamic light scattering (DLS). Zeta potential was measured following dilution in deionized water using a Zetasizer Ultra ZS (Malvern Instruments).

### Lipid Coating and Poly­(ethylene glycol)-Oligolysine Coating of the DONs

For lipid coating, DON stock solutions at 30 nM were diluted in DMEM to the target working concentration. Immediately prior to incubation, the appropriate amount of liposomes (prepared at 1 mg/mL and diluted when required in 0.1× FoB to maintain vesicle stability) was added to the DON mixture. Samples were mixed thoroughly to ensure homogeneous lipid deposition and incubated at room temperature for 30 min with an added mechanical stress of 300 rpm. The total DNA and lipid concentrations were kept constant for lipid coating formulations with varying lipid compositions.

For PEG coating, DON stock solutions of 30 nM were mixed with a 17.01 μM solution of poly­(ethylene glycol) (PEG)-oligolysine (K10–5kPEG) (Alamanda Polymers) at a 1:2 (v/v) ratio, resulting in a final N/P ratio of 0.75:1 (nitrogen atoms in amine groups to phosphate groups in DNA). The mixtures were incubated at room temperature for 30 min. Noncoated DON controls were diluted equivalently in the same medium composition.

### Lipid Nanoparticle (LNP) Formulation

RNA-loaded lipid nanoparticles (RNA-LNPs) were prepared by rapid microfluidic mixing using a NanoAssemblr Ignite system (Precision Nanosystems Inc.), as previously described.^55^ Briefly, an ethanolic lipid phase containing D-Lin-MC3-DMA, cholesterol, DSPC, and DSPE-PEG2000 at a molar ratio of 50:38.5:10:1.5 was mixed with an aqueous RNA solution in 25 mM acetate buffer (pH 4.0) at a 1:3 (v/v) organic-to-aqueous ratio. The total flow rate was set to 12 mL/min. Formulations were prepared using total lipid concentrations of 8 mM and N/P ratios of 6 (N: ionizable amine nitrogens; P: RNA phosphates). Following assembly, LNP suspensions were dialyzed against a 500-fold excess of PBS (pH 7.4) for 24 h at room temperature using 10 kDa Float-A-Lyzer dialysis cassettes to remove residual ethanol. Samples were subsequently sterile-filtered (0.2 μm) and stored at 4 °C until use.

For physicochemical characterization, LNPs were diluted 1:50 in 1× PBS for hydrodynamic size and polydispersity index (PDI) measurements, and diluted equivalently in double-distilled water (pH 7.4) for zeta potential analysis. RNA encapsulation efficiency was quantified using the Quant-iT RiboGreen RNA assay (Thermo Fisher Scientific). RiboGreen reagent was added according to the manufacturer’s instructions, and fluorescence was recorded on a SpectraMax iD5 microplate reader (Molecular Devices) at λ*_ex_* = 480 nm and λ*_em_* = 520 nm. Encapsulation efficiency (EE%) was calculated as:

$$\mathrm{EE}\%=1-\left(\frac{\mathrm{unencapsulated RNA}}{\mathrm{total RNA}}\right)\times 100\%$$

### Electrostatics Modeling

Electrostatic potential calculations were performed using a custom script implementing the linearized Poisson–Boltzmann (Debye–Hückel) equation. The DON was modeled as a uniformly charged disk (60 nm diameter, 7 nm height) with 14,000 phosphate groups. Ionic strength was set to 0.074 M for FoB and 0.131 M for 1:3 (v/v) FoB:DMEM conditions.

The Debye screening length λ_*D*_ was calculated using:

$$\lambda_{D}=\sqrt{\frac{\varepsilon \varepsilon_{0}k_{B}T}{2N_{A}e^{2}I}}$$

where *ε* = 78.5, *ε*_0_ is the vacuum permittivity, *k*_B_ is the Boltzmann constant, *T* = 298 K, *N*_A_ is Avogadro’s number, *e* is the elementary charge, and *I* is the ionic strength.

The surface potential ψ­(*z*) at distance *z* from the disk surface was calculated by:

$$\psi \left(z\right)=\frac{\sigma }{2\varepsilon \varepsilon_{0}k}e^{-kz}$$

with σ as the surface charge density and k=1λD

The electrostatically accessible fraction of phosphates was estimated as:

$$f_{\mathrm{accessible}}=\frac{2t}{h}$$

where *t* = Debye shell thickness and *h* = DON height.

The effective surface charge was scaled accordingly, and potential profiles were evaluated along the *z*-axis.

Visualization was performed using the ggplot2 and ggforce packages in R.

### Transmission Electron Microscopy

CF400-Cu grids (Electron Microscopy Sciences) were used. A total of 8 μL of the corresponding 5 nM DON solution in a 1:3 FoB:DMEM (v/v) buffer was applied to each grid and incubated for 60 s. Excess liquid was removed by gentle blotting with filter paper. Negative staining was performed by applying 1.5 μL of 2% (w/v) uranyl acetate. The stain was immediately blotted off, and the grids were air-dried at room temperature. Imaging was conducted on a Talos L120C transmission electron microscope operated at 80 kV.

### Analytical Agarose Gel Electrophoresis (AGE) for Folding, Coating Efficiency, and Stability Analysis

Folding quality and coating-induced immobilization of DON samples were evaluated by analytical agarose gel electrophoresis (AGE). A 1 kb DNA ladder (New England Biolabs) was included as a molecular weight reference. For each sample, 5 μL of DON suspension at 10 nM was mixed with 6× MassRuler loading dye (Thermo Scientific) and loaded onto a 1% (w/v) agarose gel prepared in 0.5× TBE supplemented with 8 mM MgCl_2_ and 1× SYBR Safe (Invitrogen). Electrophoresis was performed at 70 V for 90 min in an ice bath to preserve nanoparticle integrity. Gels were imaged using a ChemiDoc MP imaging system (Bio-Rad). The stability assessment of the differently coated DONs was done by exposing the particles to standard cell culture conditions or DNase I-enriched solutions. Coated DONs were diluted to a final concentration of 5 nM in the designated volume by adding either DMEM supplemented with fetal bovine serum (FBS, PAN-Biotech) to obtain a final serum concentration of 10%, or DMEM containing DNase I. DNase I working solutions were prepared by diluting the initial 1 U/mL stock solution (Thermo Scientific) in DMEM to reach the target concentrations. DON mixtures were incubated for the indicated durations at 37 °C to allow nuclease digestion.

### Dynamic Light Scattering (DLS) Characterization

For high-throughput screening, a total sample volume of 30 μL at 5 nM was prepared following the lipid coating technique explained above, with liposomes and DNA origami nanoparticles without fluorescent probes. Two microliters of each sample was loaded into the microfluidic channels of Stunner microfluidics plates and analyzed using the Unchained Laboratories Stunner DLS system. For individual hydrodynamic size measurements, 100 μL at 5 nM of each sample was prepared and analyzed using a Zetasizer Ultra ZS (Malvern Instruments).

### Nanoflow Cytometry (NanoFCM) Characterization

Samples were analyzed by nanoflow cytometry (NanoFCM). Liposomes with NBD-conjugated lipids, DNA origami nanostructures (DONs) with 6 Cy5-conjugated incorporated strands, and lipid-coated DONs presenting both fluorophores were prepared as described in the corresponding Methods sections. Stock solutions were adjusted to 5 nM DONs and/or 0.025 mg mL^–1^ liposomes, diluted 1:10 in a 1:3 Fob:DMEM (v/v) buffer, and acquired on the NanoFCM using the FITC and PC5 laser channels.

### Cell Culture

BV2 microglial cells and luciferase-expressing BV2 cells (Luc-BV2; generated by Prof. Thomas Michiels’ group, de Duve Institute, UCLouvain) were cultured in high-glucose DMEM supplemented with 10% FBS, 1% (v/v) penicillin, and 1% (v/v) streptomycin. Cells were maintained under standard conditions (37 °C, 5% CO_2_). Primary mixed glial cultures (MGCs) were extracted from Sprague–Dawley rat pups at postnatal days 1–2. Briefly, brains were dissected, and the cerebellum, olfactory bulbs, and meninges were removed. Tissue was enzymatically digested with papain (40 μg/mL) and mechanically dissociated using G18 and G23 syringe needles. The resulting cell suspension was diluted in DMEM (4.5 g/L glucose, l-glutamine, pyruvate) supplemented with 10% FBS (v/v) and 1% P/S (v/v), filtered through a 40 μm cell strainer, and cultured for 8 days prior to treatment. MGCs were maintained under the same standard conditions as above (37 °C, 5% CO_2_, 95% relative humidity).

### Cellular Uptake Quantification

BV2 cells were seeded at a density of 3.5 × 10^4^ cells per well in 48-well plates for flow cytometry, or in 8-well chambered microscopy slides (Ibidi GmbH) for confocal imaging, and incubated overnight. Cells were then treated with 150 μL of noncoated (NCD), PEG-coated (PCD), lipid-coated DON (LCD) (A647-labeled incorporated DNA strands), or LNPs (Cy5-labeled RNA) at the concentrations and incubation times specified for each experiment.

For flow cytometry acquisition, cells were detached using Accutase and transferred to V-bottom 96-well plates. Cells were washed twice with warm PBS and subsequently incubated with 80 U/mL DNase I for 1 h at 37 °C to remove extracellular nanoparticle-associated fluorescence. After nuclease treatment, cells were washed three times with cold PBS, resuspended in 120 μL FACS buffer, and analyzed using a Cytek Aurora flow cytometer.

For confocal imaging, cells were subjected to the same nuclease treatment described above, followed by staining with Hoechst and LysoTracker Green for 30 min at 37 °C. Images were acquired using a confocal microscope (brand). Manders’ and Pearson’s coefficient were determined with ImageJ Macro’s. Cellular uptake and endosomal escape were additionally assessed in primary MGCs using the same experimental procedures described above. Prior to flow cytometry acquisition, MGCs were stained with antibodies against CD11b, GFAP, and myelin basic protein (MBP) to distinguish microglia, astrocytes, and oligodendrocyte precursors, respectively, enabling cell-type-specific analysis of nanoparticle uptake within the mixed culture.

### siRNA Loading and Reductive Release Assay

siRNA functionalization and release from DNA origami nanostructures (DONs) were assessed by agarose gel electrophoresis. DONs bearing extended handle strands were hybridized with siRNA via a complementary antihandle strand containing a cleavable disulfide linkage. For loading analysis, a Cy5-labeled antihandle strand was used to enable fluorescence-based detection. For release studies, samples were incubated in the presence of 10 mM glutathione (GSH) to mimic intracellular reducing conditions. Reductive strand displacement was monitored using an Alexa Fluor 488 (A488)-labeled guide strand complementary to the antisense siRNA strand. Electrophoresis was performed on a 1% agarose gel to acquire the signal. To quantify siRNA loading stoichiometry, qPCR analysis was performed on DONs presenting 0, 10, 22, 32, 44, 56, and 72 surface handles. A standard curve was established using known concentrations of the antihandle sequence to enable absolute quantification of loaded siRNA per condition. To normalize for handle number across conditions, the DON concentration was adjusted such that a constant total antihandle of 32 nM was maintained in each qPCR reaction, allowing direct comparison of siRNA loading across DON constructs presenting different handle densities.

### Luciferase Knockdown Assay

Luc-BV2 cells were seeded in 96-well plates at a density of 1 × 10^4^ cells per well and incubated overnight. Cells were then treated with the respective nanoparticle formulations loaded with 40 nM siRNA targeting luciferase, diluted in 100 μL Opti-MEM. After 24 h of incubation, 100 μL of ONE-Glo Luciferase Assay reagent (Promega) was added to each well. Following a 10-min incubation at room temperature, luminescence was measured using a microplate reader to quantify luciferase activity.

### Cell Viability Assay (PrestoBlue)

Cell viability was assessed using the PrestoBlue assay (Thermo Fisher Scientific). BV2 cells were seeded in 48-well plates at a density of 1 × 10^5^ cells per well and incubated overnight to allow adherence. The cells were then treated with the respective formulations loaded with 40 nM antiluciferase siRNA and incubated for 24 h at 37 °C. Following treatment, the supernatant was removed. and the cells were incubated with 200 μL of PrestoBlue reagent diluted to the working concentration in culture medium. After 2 h of incubation, the supernatant was transferred to a black 96-well plate, and absorbance was measured at 550 nm using a microplate reader.

### RNA Extraction, cDNA Synthesis, and qPCR Analysis

Total RNA was extracted using TRIzol reagent (Thermo Fisher Scientific). Cells were cultured at a density of 1 × 10^5^ cells per well, treated with the different formulations loaded with 40 nM antiluciferase siRNA, and incubated for 24 h at 37 °C. Subsequently, the cells were lysed directly in 200 μL TRIzol, incubated for 5 min at room temperature, and transferred to microcentrifuge tubes. 40 μL of chloroform was added, samples were vortexed for 15 s, incubated for 5 min, and centrifuged at 12,000 × g for 15 min at 4 °C. The aqueous phase was transferred to new tubes containing 100 μL isopropanol, mixed briefly, incubated for 2–5 min, and centrifuged at 12,000 × g for 10 min at 4 °C. Pellets were washed with 200 μL of 75% ethanol, centrifuged for 10 min, air-dried for 20–30 min, and resuspended in 10 μL TE buffer. RNA was placed on ice until use or stored at −80 °C. RNA purity and concentration were measured on a NanoDrop 2000 spectrophotometer (Thermo Scientific). RNA was diluted to 1 μg in 3 μL TE for cDNA synthesis. cDNA was synthesized using the GoScript Reverse Transcription Kit (Promega) according to the manufacturer’s protocol. Reactions were incubated using a standard RT program (GoScript RT-Lente) and stored at −20 ^◦^C. qPCR reactions for mRNA targets were performed using GoTaq qPCR Master Mix (Promega). All reactions were run on a QIAquant 96 2plex instrument (Qiagen). Relative gene expression was calculated using the 2^–ΔΔ*CT*^ method. mRNA expression levels were normalized to RPL19 (mouse). Primer sequences are provided in Table S2.

### Statistical Analysis

Data analyses were performed using GraphPad Prism 10 (GraphPad Software). One-way analysis of variance (ANOVA) with Tukey’s post hoc correction for multiple comparisons, or Student’s *t*-test for pairwise comparisons, was used to determine statistical significance.

## Supplementary Material



## Notes

Additional data on DNA origami folding, lipid preparation, and optimization of lipid-coated DON formulations are provided, including DLS, nanoFCM, agarose gel electrophoresis, TEM, electrostatic modeling, flow cytometry gating, confocal microscopy, stability assays, and siRNA functional validation; and tables list CadNano handle sequences, and primer and siRNA sequences used in this study (PDF)
