# Supplementary material for: Tunable Lipid Coatings Enable Cytoplasmic siRNA Delivery by DNA Origami
Source: ACS Appl Mater Interfaces. 2026 Jun 17;18(25):34778–89. doi: 10.1021/acsami.6c03188 (PMC13339022; doi:10.1021/acsami.6c03188)
Supplement: Supplementary file 1 [file am6c03188_si_001.pdf]

SUPPORTING INFORMATION

for

Tunable Lipid Coatings Enable Cytoplasmic siRNA Delivery by DNA Origami

Pauline B. M. Hendrickx[1,2], Anne des Rieux*[1], and Maartje M. C. Bastings*[2]

[1]Advanced Drug Delivery and Biomaterials, Louvain Drug Research Institute, Université Catholique de Louvain, Brussels 1200, Belgium.

[2]Programmable Biomaterials Laboratory, Institute of Materials, Interfaculty Bioengineering Institute, School of Engineering, Ecole Polytechnique Fédérale Lausanne, Lausanne 1015, Switzerland

*Email: anne.desrieux@uclouvain.be, maartje.bastings@epfl.ch

List of Figures

Figure S1. Characterization of DNA origami folding 3

Figure S2. Liposome preparation and physicochemical characterization 3

Figure S3. High-throughput DLS screening of DNA–to–lipid ratios 4

Figure S4. Iterative optimization of lipid coating efficiency with NanoFCM 4

Figure S5. Neutralization of DON surface charge upon lipid coating 5

Figure S6. Hydrodynamic size distributions of DONs, liposomes, and lipid-coated DONs 5

Figure S7. Electrostatic modelling of the DON 6

Figure S8. TEM images of pCat LCDs 6

Figure S9. Influence of mol% cationic lipid on the size and charge neutralization of lipid-coated DON 7

Figure S10. Flow cytometry gating strategy and nuclease sensitivity of differently coated DONs 7

Figure S11. Confocal images of pCat LCDs 8

Figure S12. Dose-response and kinetics stability test of differently coated DONs 8

Figure S13. Cellular uptake and endosomal escape of DONs and LNPs in primary mixed glial cultures 8

Figure S14. siRNA functionalization onto DONs 9

Figure S15. Negative control assay for luciferase knockdown 9

Figure S16. Inflammation resulting from pCat LCD incubation 10

List of Tables

Table S1. CadNano handle sequences 11

Table S2. Primers and siRNA sequences 12

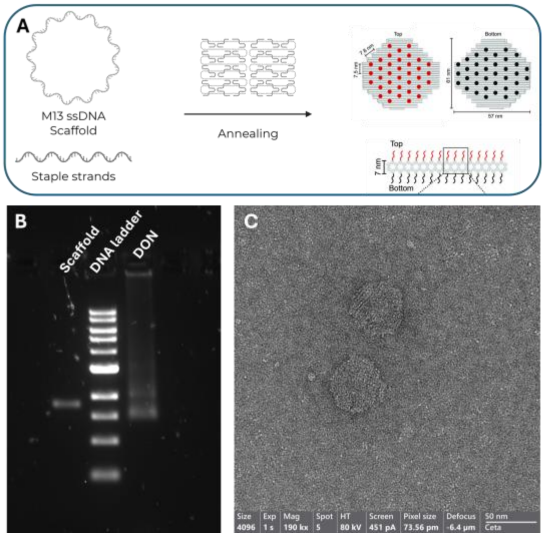

Figure S 1: Characterization of DNA origami folding and functional handle incorporation. (A) Schematic representation of the folding of the p7560-based DNA origami disk and location of protruding staple extensions used for functionalization. (B) Verification of successful folding by 1% agarose gel electrophoresis, showing migration of the assembled structure relative to the scaffold strand, and (C) corresponding TEM image confirming expected disk-like morphology.

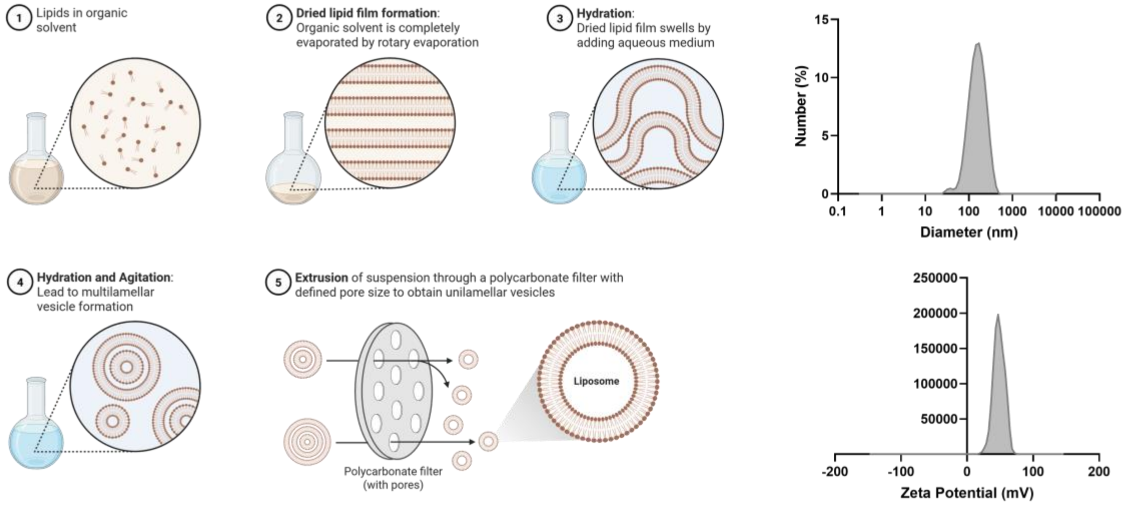

Figure S 2: Liposome preparation and physicochemical characterization. (A) Schematic overview of the lipid film hydration and extrusion process used to generate unilamellar liposomes. Lipid mixtures dissolved in organic solvent were dried to form a thin lipid film, rehydrated to produce multilamellar vesicles, and extruded through a polycarbonate membrane with defined pore size to obtain monodisperse unilamellar liposomes. Created in BioRender. Desrieux, A. (2026) https://BioRender.com/nxw575t (B) Representative size distribution and zeta potential measurements of 1:1 mol% DOPC:DOTAP liposomes, confirming monodispersity and expected surface charge characteristics.

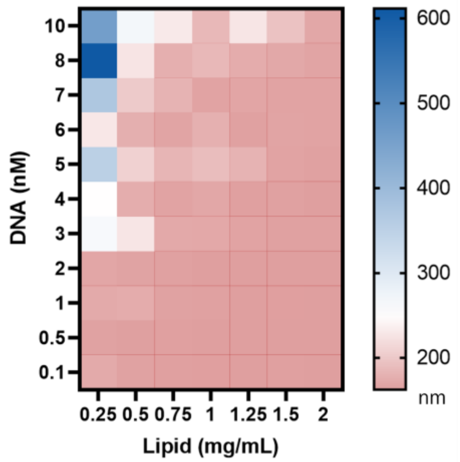

Figure S 3: High-throughput DLS screening of DNA–to–lipid ratios. Heatmap showing hydrodynamic size distributions obtained by high-throughput DLS as a function of DON concentration and lipid concentration. Increased particle sizes (blue) indicate lipid-induced aggregation at elevated lipid inputs or higher DON concentrations, whereas lower sizes (red) correspond to monodisperse formulations. The screening was used to identify the formulation window minimizing aggregation during lipid coating.

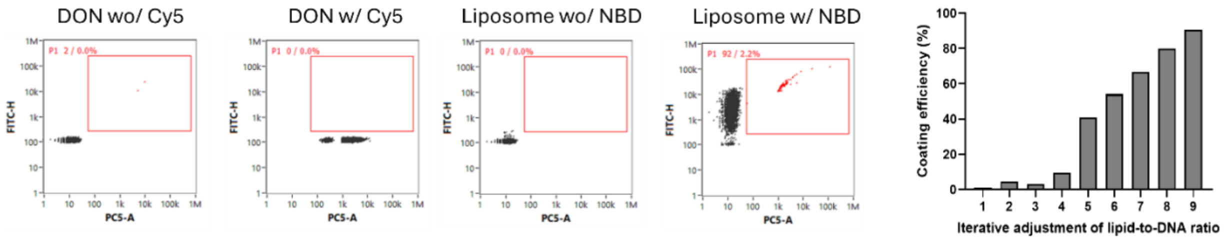

Figure S 4: The gating strategy for the Nano-FCM analysis and the following iterative optimization of lipid coating efficiency. NanoFCM colocalization analysis of Cy5-labeled DONs and NBD-labeled lipids across sequential formulation adjustments. Refinement of the lipid-to-DNA ratio progressively increased the fraction of dual-positive particles from less then 10% to 90%, indicating successful improvement of lipid-coating efficiency. The iteration being: 1) 1 nM DNA and 2 mg/mL lipid 2) 5 nM DNA and 1 mg/mL lipid 3) 8 nM DNA and 0.5 mg/mL lipid 4) 8 nM and 2 mg/mL lipid 5) 2 nM DNA and 0.05 mg/mL lipid 6) 10 nM DNA and 0.025 mg/mL 7) 10 mM DNA and 0.1 mg/mL lipid 8) 5 nM DNA and 0.01 mg/mL lipid 9) 5 nM DNA and 0.025 mg/mL lipid.

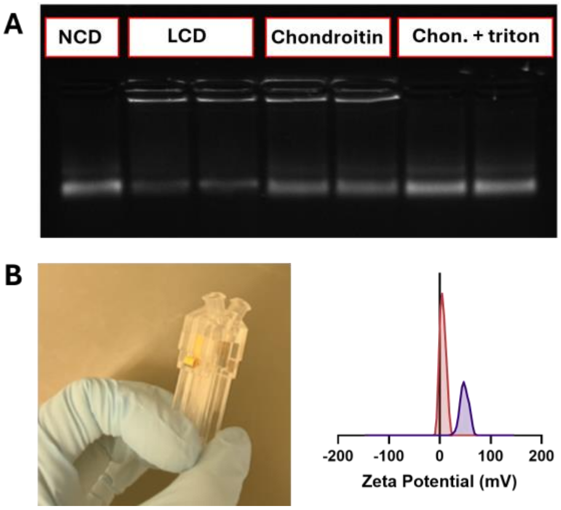

Figure S 5: Neutralization of DON surface charge upon lipid coating. (A) Analytical 1% agarose gel demonstrating charge-dependent immobilization of DONs. Bare DONs migrate into the gel, whereas lipid-coated DONs remain trapped in the well due to electrostatic charge neutralization. Treatment with chondroitin sulfate, and subsequently with chondroitin plus Triton X-100, restores migration, confirming disruption of lipid–DNA electrostatic interactions. (B) Photograph of damaged electrodes resulting from attempted zeta potential measurement of non-coated DONs, illustrating their strong attraction to the electrode surface. In contrast, lipid-coated DONs yield stable zeta potential measurements (red), consistent with surface charge neutralization, while free liposomes exhibit a higher positive zeta potential (purple).

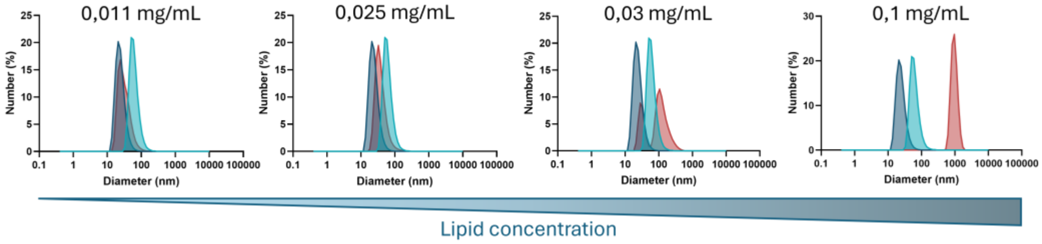

Figure S 6: Hydrodynamic size distributions of DONs, liposomes, and lipid-coated DONs. Dynamic light scattering (DLS) measurements comparing the size distributions of bare DONs (dark blue), 1:1 DOPC:DOTAP liposomes (light blue), and lipid-coated DONs prepared with a constant DNA concentration (5nM) and increasing lipid concentrations. Progressive right-shifts in the distribution profiles indicate increasing hydrodynamic size with higher lipid content, consistent with formation of lipid-coated DONs and, at high lipid excess, early signs of lipid-induced aggregation.

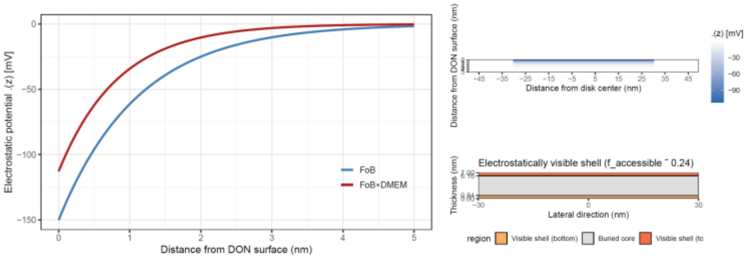

Figure S 7: Electrostatic potential distribution around the DON surface and estimation of the electrostatically accessible phosphate fraction. (A) Electrostatic potential $\psi(z)$ as a function of distance from the DON surface, calculated under FoB and FoB × DMEM ionic conditions using a linearized Poisson–Boltzmann model. Increased ionic

strength (FoB × DMEM) results in stronger screening and a more rapid decay of the surface potential. (B) Cross-sectional visualization of the potential profile across the DON, showing lateral decay of $\psi(z)$ from the disk center. (C) Schematic representation of the electrostatically visible outer shell of the DON, defined by the Debye screening length under physiological salt conditions. Only phosphate groups located within this ~ 1 nm outer shell contribute to long-range electrostatic interactions.
The accessible fraction of phosphates is estimated as $f_{\text{accessible}} \approx 0.24$, consistent with the reduced effective charge density observed during lipid coating.

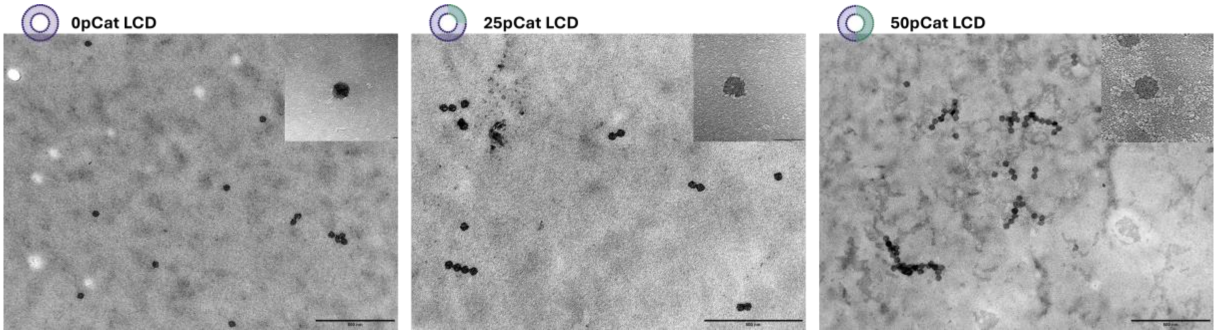

Figure S 8: TEM image of lipid-coated DONs with increasing DOTAP inclusion. Scale bar: 500 nm (zoom out) and 50 nm (zoom in)

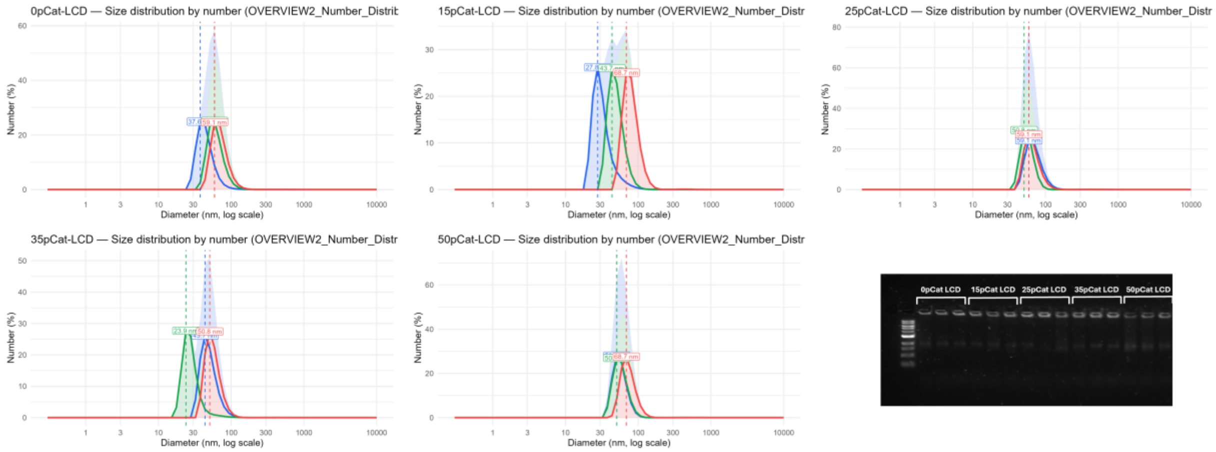

Figure S 9: Hydrodynamic size and charge-neutralization behavior of lipid-coated DONs with increasing DOTAP content. The total DNA and lipid concentration were kept constant for the lipid coating formulations with varying lipid compositions. Dynamic light scattering (DLS) measurements showing hydrodynamic size distributions of DONs coated with liposomes containing 0, 15, 25, 35, and 50 mol% DOTAP. The accompanying 1% agarose gel demonstrates DOTAP-independent charge neutralization. (N=3)

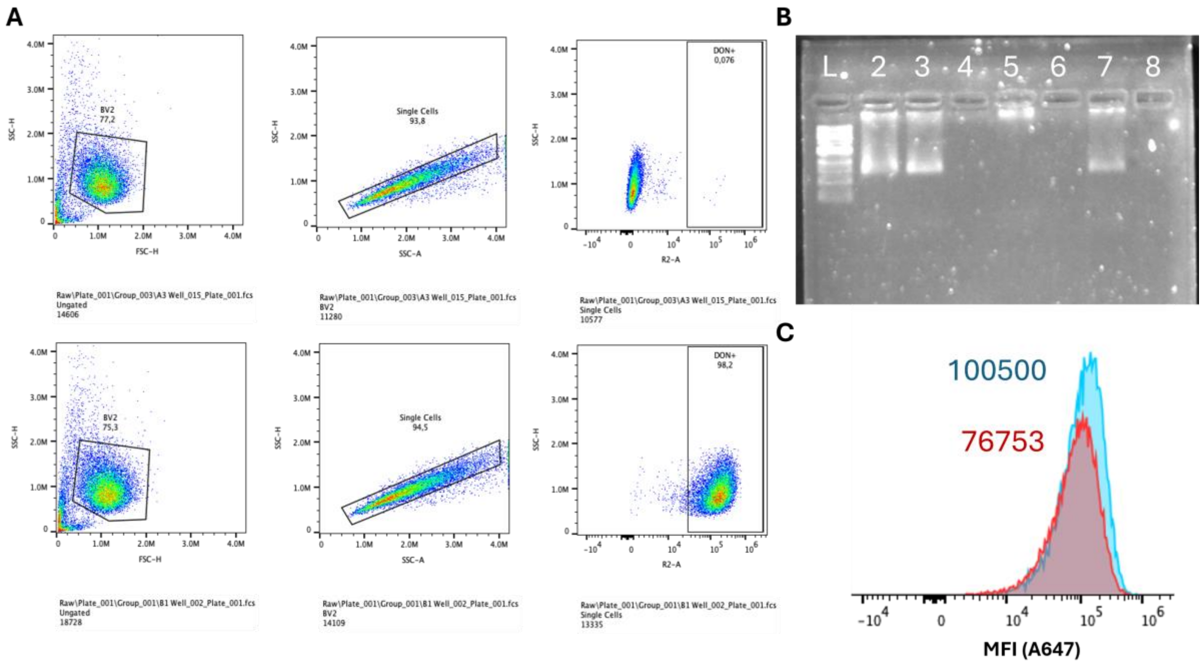

Figure S 10: Flow cytometry gating strategy and nuclease sensitivity of differently coated DONs. (A) Gating strategy for BV2 microglial cells used to quantify cellular internalization of A647-labeled DONs. Single-cell populations were identified using FSC/SSC parameters, followed by gating on A647-positive events. (B) Agarose gel (1%) showing nuclease sensitivity of DONs with different coatings following treatment with 80 U/mL DNase I for 1 h after being diluted in standard cell culturing conditions for 12h. Lanes: 1 = ladder; 2 = DON; 3 = NCD (12 h diluted); 4 = NCD + DNase; 5 = PCD (12 h diluted); 6 = PCD + DNase; 7 = LCD (12 h diluted); 8 = LCD + DNase. (C) Mean fluorescence intensity (MFI) of A647-labeled DONs measured by flow cytometry before (blue) and after (red) 80 U/mL DNase I treatment following 12 h incubation with BV2 cells.

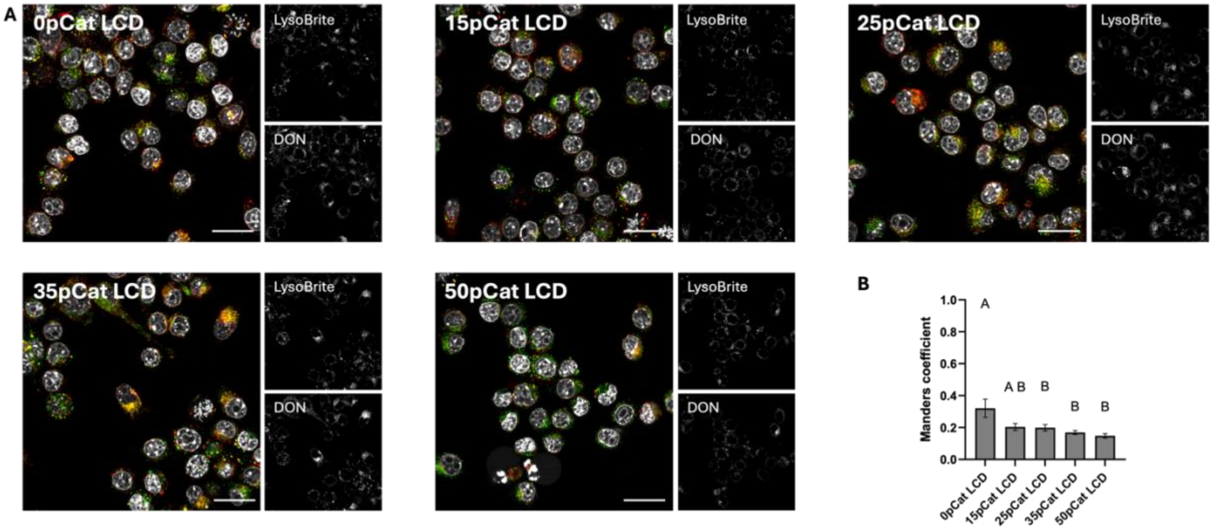

Figure S 11: Cellular uptake and endosomal retention of lipid-coated DONs with varying lipid compositions. **(A)** Confocal fluorescence microscopy images showing the internalization of LCDs in BV2 microglial cells after 6 h of incubation. DONs were visualized through A647-labeled DNA (red), nuclei stained with Hoechst (blue), and acidic compartments stained with Lysotracker Green (green). **(B)** Endosomal retention quantified from confocal images

with Lysotracker Green staining and A647-conjugated DONs. Mander's coefficient quantifies the overall overlap of Lysotracker Green and A647 (N=2). Statistical analysis: One-way ANOVA followed by Tukey's post-hoc test. Conditions not sharing the same letters are significantly different.

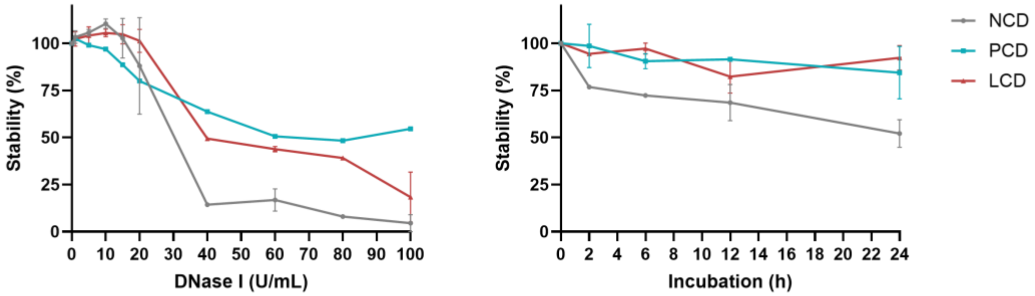

Figure S 12: Stability of NCD, PCD, and LCD under nuclease challenge and serum-containing conditions. (Left) Dose-response nuclease stability. DONs (5 nM) coated with NCD, PCD, or LCD were incubated with increasing concentrations of DNase I (0–100 U/mL) for 2 h at 37 °C. Stability was quantified by analysis of 1% agarose gel electrophoresis band intensity and expressed relative to untreated controls. (Right) Time-dependent serum stability. DONs (5 nM) were incubated in medium containing 10% FBS for up to 24 h, and structural integrity was assessed by AGE band intensity quantification and expressed relative to untreated controls. (N=2)

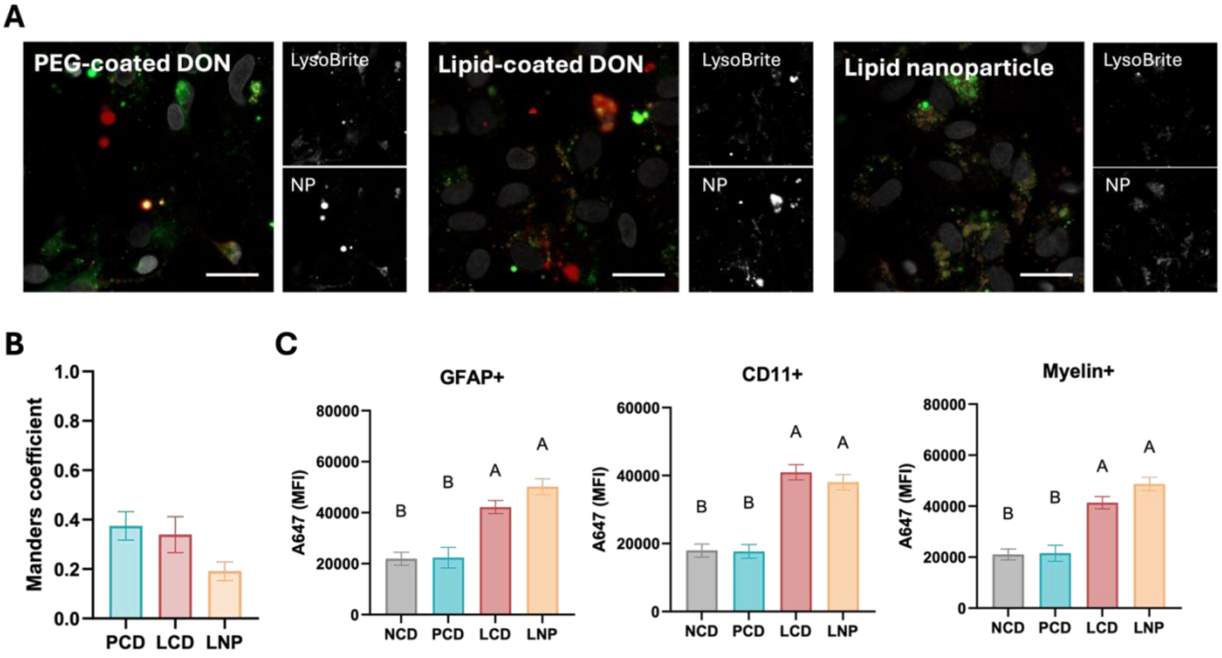

Figure S 13: Cellular uptake and endosomal escape of DON formulations and LNPs in primary mixed glial cultures. **(A)** Representative confocal microscopy images of MGCs following 24h incubation with PEG-coated DONs (PCDs), lipid-coated DONs (LCDs), or LNPs (all shown in red), counterstained with LysoBrite to visualise lysosomal compartments (green). Scale bars indicate 50 µm. **(B)** Quantification of Manders coefficient reflecting the degree of co-localisation between nanoparticle signal and lysosomal compartments. **(C)** Quantitative flow cytometry analysis of cellular uptake following 24h incubation with each formulation. Prior to acquisition, MGCs were stained with antibodies against CD11b, GFAP, and MBP to enable cell-type-specific quantification of nanoparticle uptake in microglia, astrocytes, and oligodendrocyte precursors, respectively. (N=2, n=4) One-Way ANOVA, Tukey's post-hoc.

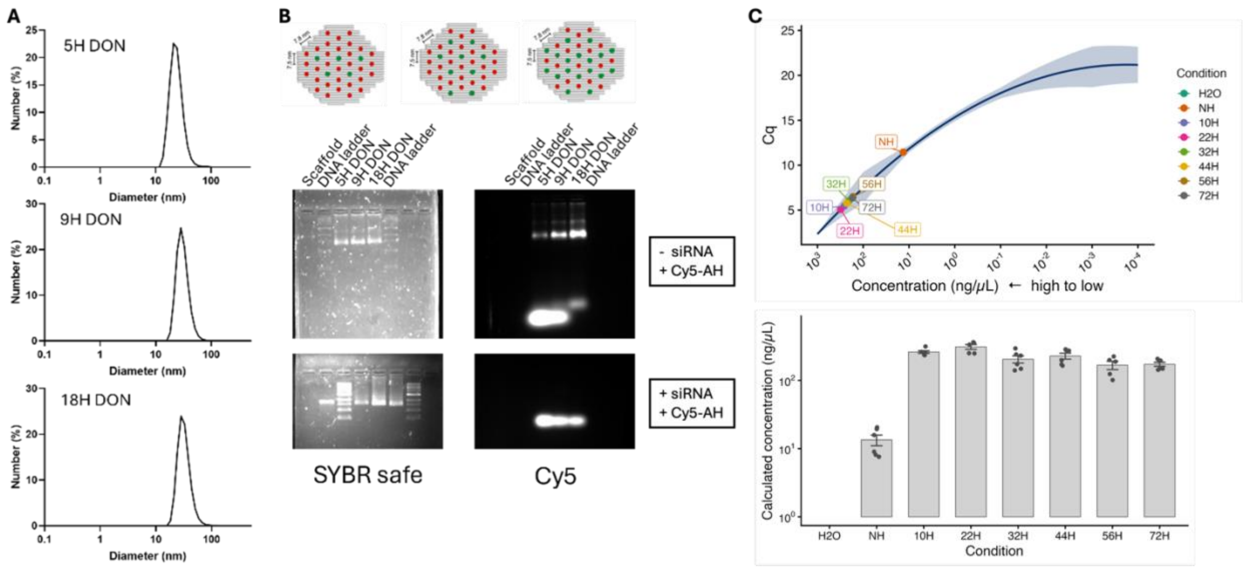

Figure S 14: Characterisation of siRNA loading on DONs with varying handle densities. **(A)** Hydrodynamic radius of siRNA-loaded LCDs across handle conditions. **(B)** Qualitative assessment of siRNA loading capacity by competitive displacement assay. DONs presenting 5, 9, or 18 surface handles were incubated with siRNA, followed by addition of Cy5-labelled anti-handle strands (Cy5-AH) as reporters for unoccupied binding sites. Strong Cy5 signal indicates residual accessible handles, while loss of signal reflects successful siRNA occupancy. Total DON content was visualised by SYBR Safe staining and Cy5 fluorescence was used to assess handle availability on 1% agarose gels. **(C)** Quantitative siRNA loading efficiency determined by qPCR for DONs presenting 0, 10, 22, 32, 44, 56, and 72 surface handles. A standard curve (blue) relating anti-handle concentration to Cq values demonstrates consistent siRNA loading across all handle densities tested.

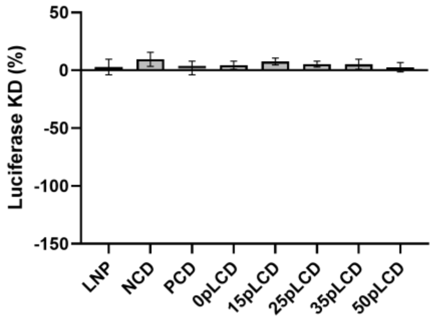

Figure S 15: Negative control assay for luciferase knockdown using non–siRNA-loaded nanoparticles.LNPs containing scrambled RNA and DON-based formulations (NCD, PCD, and LCD variants) without siRNAAH were incubated with luciferase-expressing BV2 cells for 24 h to assess the particles' effects on luciferase expression. No appreciable knockdown was observed across any non-loaded formulation, confirming that luciferase silencing in subsequent experiments arises from siRNA payload activity (N = 2).

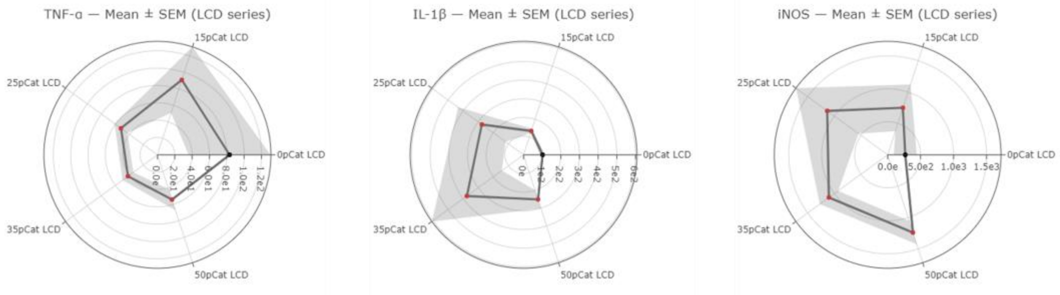

Figure S 16: Inflammatory response induced after incubation with the different nanoparticle formulations, quantified by qPCR analysis, normalized to set the Vehicle to 100 (Veh.). (N=3)

Table S 1: CadNano location of handle sequences used in this study.

CadNano position	Sequence (5′–3′)
42[160]68[149]	TTATTACATACCACGGAACGCTAAACGTGGCTGAAAATCTCCTGACATG
46[76]72[65]	TAAGCCCTAGACGGAATACATGTTTGAGGGCTGAAAATCTCCTGACATG
52[186]78[171]	AAATGTCGTCTTAATTGTAAATTCGTGGAGGATGGCTGAAAATCTCCTGACATG
50[160]76[149]	ATACCGATAAAATACTGCCATAAATAACGGCTGAAAATCTCCTGACATG
60[99]86[87]	TACTGGTAAGTTCCAGATTTAGAAAAGGAAGGCTGAAAATCTCCTGACATG
54[160]80[149]	CAGTACACCTCATAACTCACACGGAAGCGGCTGAAAATCTCCTGACATG
44[97]70[87]	AAGGCTTATTGGGCTAGATGGATGGCAAGGCTGAAAATCTCCTGACATG
50[118]76[108]	GCATAACAGGACTAGCCTTGATCCTTAGGGCTGAAAATCTCCTGACATG
51[88]78[87]	ATTACCATAGGGAAAAACATTTCTGGCTGAAAATCTCCTGACATG
56[97]82[87]	AGGCAGGACCAGAAAGAGCGGTCGGCCAGGCTGAAAATCTCCTGACATG
33[130]62[129]	CATGCTGAATGGCTTAATTGAGTTACGCAAGACATTATGGCTGAAAATCTCCTGACATG
45[109]72[108]	AGGGTAATGATTAGGAGCTCCAGCGGCTGAAAATCTCCTGACATG
38[118]64[108]	AATGACCGGAAGCCGTCAAATAGAGTCAGGCTGAAAATCTCCTGACATG
54[118]80[107]	AATCACCCGGTCATGGGAAACATCGGCCGGCTGAAAATCTCCTGACATG
52[55]78[44]	AGCACCGCATTTGGGTCTGAAACACGACGGCTGAAAATCTCCTGACATG
48[97]74[87]	ATAAGTTCGCAATAGGTGAGGAGTTGGCGGCTGAAAATCTCCTGACATG
52[139]78[128]	TTTGCTAACGTTGATATCCGCACAGGGCGGCTGAAAATCTCCTGACATG
44[181]70[171]	GCTGACCAGGACGTTTAAATGTTCCTGTGGCTGAAAATCTCCTGACATG
60[144]86[127]	AAACCCCCTGCCGTATTAAGGAACAAATAGGGTGGCTGAAAATCTCCTGACATG
36[97]62[87]	AGGCATTATTCTTAACCTCCGAATAAAGGGCTGAAAATCTCCTGACATG
38[160]64[150]	ATTGAATTCAAAGCTGTGTAGTATTTTAGGCTGAAAATCTCCTGACATG
44[55]70[44]	GATTTTTACAAAATTTTGAGTCAGAAGGGGCTGAAAATCTCCTGACATG
54[76]80[65]	AGCCGCCTGCCTTTCCACCGAATTAGTAGGCTGAAAATCTCCTGACATG
48[139]74[128]	TAAAGGTACTCCTTGTGGTTGTGCAAGGGGCTGAAAATCTCCTGACATG
46[160]72[149]	TTGTGTCCCAACTTCTATTACGGCAAAGGGCTGAAAATCTCCTGACATG
58[118]84[107]	GCTCAGTTATAAGTGCGGTCACCCAGCAGGCTGAAAATCTCCTGACATG
48[55]74[44]	TACCAGCAGCAGATCTGAGAGTTGCTGAGGCTGAAAATCTCCTGACATG
42[76]68[65]	TGCACCCTACCGCGAGATGAAAAAATCGGGCTGAAAATCTCCTGACATG
37[55]64[65]	AAAACAAAAGGTAAAGTAATGTCTTCTGTATCCTTGGGCTGAAAATCTCCTGACATG
44[139]70[129]	CATTACCATTACCTACAAACGTCTGAATGGCTGAAAATCTCCTGACATG
40[139]66[129]	GATAAAATTTTGCCAACAAGATCTAGCTGGCTGAAAATCTCCTGACATG
56[139]82[128]	TCAGAACGGGATAGAGAGTTGAGGGTGGGGCTGAAAATCTCCTGACATG
40[97]66[87]	TAGAAACGTCCTGAAATCATACTTTTTTGGCTGAAAATCTCCTGACATG
50[76]76[65]	AAGGCCGGACAGCAGACCTGAAAACATCGGCTGAAAATCTCCTGACATG
42[118]68[107]	GCGGGAGCCGGTATAACAGAAGCCCCAAGGCTGAAAATCTCCTGACATG
48[181]74[171]	AGAATACATACCAAGCCAAGCACGACGTGGCTGAAAATCTCCTGACATG
17[30]71[46]	AAAGACAACTCGTCATTTTCTAATTTACGCTAAGGCTGAAAATCTCCTGACATG
13[30]75[46]	AAATGGCTATTACAAATGATTTTAAGGCAATAGGGCTGAAAATCTCCTGACATG
9[77]79[87]	ATAACATGGAAATACCAGTAGGGAGTTAGGCTGAAAATCTCCTGACATG
15[140]73[150]	CGATTAAGGCGAAAGGGAACCGGATATTGGCTGAAAATCTCCTGACATG
17[77]71[87]	GGGACGAGTAACCGAGAACGAGCTATTTGGCTGAAAATCTCCTGACATG
13[119]75[130]	TGCTGAAATGCGCACTGGCATAGCCGGAGGCTGAAAATCTCCTGACATG
12[44]79[46]	AATACCAGTCATGGATTATTTACATCACCGAATTCATTGGCTGAAAATCTCCTGACATG
48[196]77[193]	AAAGAAAGAGGCAAAGGCTGAAAATCTCCTGACATG
54[175]83[172]	AAAGAGTTTCGTCACGGCTGAAAATCTCCTGACATG
12[128]79[130]	GAATGAGTAATCTCCAGAACAATAATAATAACGCCCACGGCTGAAAATCTCCTGACATG
7[98]81[109]	ACGCGCGGCCAGCTTTCATCGAGAATAGGGCTGAAAATCTCCTGACATG
22[149]69[150]	GTCTAAATTGCAAGAACCTACCATGTTGAGAAGACGACGGCTGAAAATCTCCTGACATG
11[140]77[150]	TTAAGCTCAACTCGTTCCATTACATACAGGCTGAAAATCTCCTGACATG

3[98]85[109]	GGGAAGACGTAACCGTCGAGAGAGGTTGGGCTGAAAATCTCCTGACATG
11[98]77[109]	CCGAACTGACGCATCTACAGACCACGGAGGCTGAAAATCTCCTGACATG
3[140]85[151]	TGAGTGTTCAAAAGACCGTACGCCACCCGGCTGAAAATCTCCTGACATG
5[119]83[130]	GGCGAAACGGTCCACACCCTCTAATCAAGGCTGAAAATCTCCTGACATG
15[98]73[109]	AAATCAAAACTAATAACAAAGAGTGAATGGCTGAAAATCTCCTGACATG
15[56]73[66]	ACCTCAATTTAGAAACAGGGAAAGAAACGGCTGAAAATCTCCTGACATG
44[196]73[193]	AAAGCGCATAGGCTGGGCTGAAAATCTCCTGACATG
17[161]71[171]	CGCCATTGTAACAATCATTATAACAACAGGCTGAAAATCTCCTGACATG
13[77]75[87]	GCCATTATATTAACGGAAACCATTGAGTGGCTGAAAATCTCCTGACATG
26[107]65[109]	TACGCTGAGACAACATAAATCAATCAAAAAGTAGGCAGGGCTGAAAATCTCCTGACATG
23[140]65[151]	GATAAATTTCAAAATTTTAATGAGAGTAGGCTGAAAATCTCCTGACATG
6[80]83[87]	GGAGCCCTCAGCCCTCAGGGCTGAAAATCTCCTGACATG
17[119]71[130]	CAGCTTTGACCGTATTTAATCCCGACTTGGCTGAAAATCTCCTGACATG
19[56]69[66]	AGCGGAATAACAGTATCGTAGCATTCCAGGCTGAAAATCTCCTGACATG
13[161]75[171]	CCCGCTTGGAGAAGGACCCCCTGATAAAGGCTGAAAATCTCCTGACATG
9[161]79[171]	ATAAAGTGTCATAGCAAAAGGCAGCTTGGGCTGAAAATCTCCTGACATG

21[119]67[130]	AAACAGGTGAACGGTTTTGCAAAACGAGGGCTGAAAATCTCCTGACATG
24[86]67[87]	AATGAATTACTGTTATTCATTTCATCAACAAACTATTAGGCTGAAAATCTCCTGACATG
21[161]67[171]	TAATATTTCAGGTCTTTAGACAATATTCGGCTGAAAATCTCCTGACATG
11[56]77[67]	CAGTAATAGAATACCCTCAGCTATGGTTGGCTGAAAATCTCCTGACATG
7[51]81[68]	AAAGGAACGGTAAGTCTGTGACAGAACGATAGCGGCTGAAAATCTCCTGACATG
7[140]81[150]	TTTTTCTCGTTGCGATTCCACATGGGATGGCTGAAAATCTCCTGACATG

Table S 2: Functional oligonucleotide sequences used in this study (primers and siRNA).

Name	Sequence (5′–3′)
Primers	
IL1b – Forward	TCGCTCAGGGTCACAAGAAA
IL1b – Reverse	CATCAGAGGCAAGGAGGAAAAC
iNOS – Forward	AGGTACTCAGCGTGCTCCAC
iNOS – Reverse	GCACCGAAGATATCTTCATG
TNFa – Forward	AGCCCCCAGTCTGTATCCTT
TNFa – Reverse	GGTCACTGTCCCAGCATCTT
RPL19 – Forward	TGACCTGGATGAGAAGGATGAG
RPL19 – Reverse	CTGTGATACATATGGCGGTCAATC
siRNA / labeled oligonucleotides	
siRNA – Guide strand	/5Phos/rUrUrUrArCrArUrArArCrCrGrGrArCrArUrArArUrCrGrUrU/S-S/CATGTCAGGAGATTTTCAGCC
siRNA – Passenger strand	rCrGrArUrUrArUrGrUrCrCrGrGrUrUrArUrGrUrArArA
Cy5-AH	Cy5-CATGTCAGGAGATTTTCAGCC
A488 – Passenger strand	CGATTATGTCCGGTTATGAAA/3AlexF488N/
